# Targeting CDH17 Suppresses Tumor Progression in Gastric Cancer by Downregulating Wnt/β-Catenin Signaling

**DOI:** 10.1371/journal.pone.0056959

**Published:** 2013-03-15

**Authors:** Hai-bo Qiu, Li-yi Zhang, Chao Ren, Zhao-lei Zeng, Wen-jing Wu, Hui-yan Luo, Zhi-wei Zhou, Rui-hua Xu

**Affiliations:** 1 State Key Laboratory of Oncology in South China, Guangzhou, Guangdong, China; 2 Department of Medical Oncology, Sun Yat-sen University Cancer Center, Guangzhou, Guangdong, China; 3 Department of Gastric and Pancreatic Surgery, Sun Yat-sen University Cancer Center, Guangzhou, Guangdong, China; 4 Department of Clinical Oncology, The University of Hong Kong, Pokfulam, Hong Kong, China; 5 Division of Surgical Oncology, Dana-Farber Cancer Institute and Harvard Medical School, Boston, Massachusetts, United States of America; Northwestern University Feinberg School of Medicine, United States of America

## Abstract

**Purpose:**

Gastric cancer remains one of the leading causes of cancer death worldwide. Patients usually present late with local invasion or metastasis, for which there are no effective therapies available. Following previous studies that identified the adhesion molecule Cadherin-17(CDH17) as a potential marker for gastric carcinoma, we performed proof-of-principle studies to develop rational therapeutic approaches targeting CDH17 for treating this disease.

**Methods:**

Immunohistochemistry was used to study the expression of CDH17 in 156 gastric carcinomas, and the relationship between survival and CDH17 expression was studied by multivariate analyses. The effect of RNA interference–mediated knockdown of CDH17 on proliferation of gastric carcinoma cell lines was examined in vitro and in vivo, as well as the effects on downstream signaling by immunoblotting.

**Results:**

CDH17 was consistently up-regulated in human gastric cancers, and overall survival in patients with CDH17 upregulation was poorer than in those without expression of this gene (5 yrs overall survival rate 29.0% vs. 45.0%, P<0.01). Functional assays demonstrated that CDH17 knockdown inhibited cell proliferation, adhesion, migration, invasion, clonogenicity and induce G0/G1 arrest. In mice, shRNA-mediated CDH17 knockdown markedly inhibits tumor growth; intratumoral injection of CDH17 shRNAs results in significant antitumor effects on transplanted tumor models. The antitumor mechanisms underlying CDH17 inhibition involve inactivation of Wnt/β-catenin signaling.

**Conclusion:**

Our results identify CDH17 as a biomarker of gastric carcinoma and attractive therapeutic target for this aggressive malignancy.

## Introduction

Gastric cancer is the fourth most common cause of cancer death in the world. Although the incidence of gastric cancer has decreased dramatically in some developed countries over the past decade, a total of one million new cases are estimated to be diagnosed each year [1]. Patients with this disease commonly have a poor outlook, accounting for 10% of total cancer deaths [2]. Even after radical surgery, more than half of the patients recur. Lymph node involvement, depth of tumor invasion and tumor location have been identified as the most significant clinicopathologic prognostic factors [3], [4].

Local invasion and distant metastasis commonly occur in advanced gastric carcinoma. The process of invasion and metastasis is complex and requires the dysregulation of a variety of cellular elements including critical adhesion molecules. Cadherin belongs to one of the adhesion molecule families and plays a crucial role in establishing cell-cell interaction [5]. Cadherin-17 (CDH17), which is also called liver–intestine (LI) cadherin or human peptide transporter-1 (HPT-1), is a structurally unique member of the cadherin superfamily [6], [7]. Whereas the so-called classic cadherins, such as E-, N- and P-cadherin, have five cadherin repeats within the extracellular domain, CDH17 consists of seven cadherin repeats. Moreover, CDH17 has only 20 amino acids in the cytoplasmic domain, whereas classic cadherins have a highly conserved cytoplasmic domain consisting of 150–160 amino acids. CDH17 is expressed in mice and humans almost exclusively in epithelial cells of both embryonic and adult small intestine and colon, with no detectable expression in the stomach and liver [8].

Utilizing integrative genomic and proteomic approaches, researchers have begun to identify novel oncogenes and tumor suppressors in gastric cancer. Previous studies of clinical cohorts identified CDH17 as a potential disease marker for gastric carcinoma [9], [10]. Despite these significant clinical findings, the molecular functions of CDH17 remain unknown, and its tumorigenic role in gastric carcinoma has not yet been confirmed. Here, we aimed to dissect the oncogenic signaling mechanisms of CDH17 in the gastric carcinoma context and evaluated the effects of targeting CDH17 as a potential therapeutic approach for gastric cancer.

## Methods

### Patients

The study population consisted of 156 consecutive patients (106 men and 50 women) scheduled for surgery from 1 January 2002 to 31 December 2006 at Sun Yat-Sen University Cancer Center, Guangzhou, China. All patients enrolled in this study had a histologically confirmed diagnosis of primary gastric carcinoma that was further analyzed pathologically after surgery. Surgery consisted of subtotal or total gastrectomy in all patients; a standardized technique was used for surgical resection and lymphadenectomy, as described elsewhere [3]. Patients who underwent non-resective surgery, and those with Siewert type I cardia adenocarcinoma were excluded from the study. Specimen and tumor sizes were recorded by the pathologists. Representative areas of the tumor were selected and snap-frozen. pTNM classification followed the criteria of the 6th edition of the UICC [11].The median age of the patients was 57.2 yrs (range: 27–78 yrs)Age, sex, type of surgery, tumor location, TNM stage, chemotherapy, specimen length, tumor size, and histological differentiation, were recorded for each patient in a database. All the patients who underwent any type of chemotherapy were on the 5-FU, platinum or taxol-based regimens. All patients, after discharge from the hospital, entered a follow-up program according to standard protocol [12]. The follow-up was closed in April 2010. The study was approved by the ethics committee in Sun Yat-sen University Cancer Center, all participants provided their written informed consent to participate in this study.

### Cell Lines and CDH17 antibody

Gastric carcinoma cell lines, AGS, MKN-45, HGC-27, MGC-803 BGC-823 and SGC-7901 were obtained from the American Type Culture Collection (Manassas, VA), Japanese Cancer Research Resources Bank (Tokyo, Japan) and Beijing Medical University (Beijing, China). Cells were cultured in RPMI 1640 medium (Sigma), supplemented with 10% fetal bovine serum (Invitrogen) and 1% penicillin-streptomycin (Invitrogen). A mouse monoclonal antibody to CDH17 (ab54511) was from Abcam (Cambridge, MA).

### CDH17 short hairpin RNA (shRNA)

The pLKO.1 puro (7 kb) lentiviral construct contains a U6 promoter and HIV-1 RNA packaging signal with pruomycin- and ampicillin- resistance elements cloned 3′ of the human phosphoglycerate kinase (hPGK) promoter. A cpptCTE was inserted 5′ of the hPGK promoter. Human CDH17 shRNA constructs were generated by ligating the following annealed oligomers into the unique Agel and EcoRI sites of pLKO.1 puro: CDH17 shRNA forward (5′- CCG GCC ACT TTC ATA TCC GCT GGA ACT CGA GTT CCA GCG GAT ATG AAA GTG GTT TTT G -3′) and reverse (5′- AAT TCA AAA ACC ACT TTC ATA TCC GCT GGA ACT CGA GTT CCA GCG GAT ATG AAA GTG G -3′). Lentiviral preparations were produced by co-transfecting pLKO.1 puro empty vector with CDH17 shRNA, and helper virus packaging plasmids pCMVΔR8.9 and pMG.G (at a 10∶10∶1 ratio) into 293T cells. Transfection was performed using lipofectamine and PLUS reagent (Invitrogen). Lentiviruses were harvested at 24, 36, 48 and 60 h after transfection. Two controls were set up, in which the gastric cancer cells either received no treatment, or were transfected with scrambled non-targeted RNAi vector (Mock). Infection was carried out in the presence of 8 µg/ml of polybrene. Following infection, AGS and MKN-45 cells were selected for stable expression of the shRNAs using 2 µg/mL puromycin. Cells were lysed for western blot analysis 10 days post-infection.

### Immunoblotting and subcellular fractionation

Protein lysates were prepared from cell line monolayers using lysis buffer (1% NP-40,50 mM Tris-HCl, pH 8.0, 100 mM sodium fluoride, 30 mM sodium pyrophosphate, 2 mM sodium molybdate, 5 mM EDTA, 2 mM sodium orthovanadate) containing protease inhibitors (10 mg/ml aprotinin, 10 mg/ml leupeptin, 1 mM phenylmethylsulfonyl fluoride). Protein concentrations were determined using the Bio-Rad protein assay (Bio-Rad Laboratories Hercules, CA, USA). Cell lysates were subjected to sodium dodecyl sulfacte-polyacrylamide gel electrophoresis (SDS-PAGE), and the separated proteins were electrophoretically transferred to immobilon-P membranes (Millipore). After blocking by non-fat milk, the membranes were incubated independently in primary antibodies P53 (Cell Signaling #9282), MDM2 (Zymed #33-7100), P21 (Zymed #33-7000), Cyclin D1 (Santa Cruz sc-753), Rb (Cell Signaling #9309), β-Catenin (Zymed, 13-8400), GSK3β (Santa Cruz, sz-7291) and p-GSK3β (Ser9) (Cell Signaling #9336). The membrane was washed several times in PBS with 0.1% Tween20 and scanned in the LI-COR Odyssey infrared two-color detection imager system (LI-COR Bioscience) according to the manufacturer's instructions.

Subcellular fractionations were performed using specific cell lysis buffers and repeated centrifugation steps (Nuclear extract kit from Active Motive, Carlsbad, CA) according to the manufacturer's specifications. Cell fractions were dissolved in RIPA buffer, and the protein concentration was measured using BCA protein quantification assay. Protein extracts (20 µg) were further dissolved in 1× Laemmli buffer, subjected to electrophoresis on a 10% SDS-PAGE and transferred to nitrocellulose membranes as described above. Western blot analysis of poly-ADP-ribose polymerase (PARP, from Cell Signaling Technology Inc, Beverly, MA) and superoxide dismutase 2 (SOD2, from ABFrontier, Seoul, Korea) were performed to confirm appropriate separation of nuclear and cytosolic proteins.

### Quantitative reverse transcription-PCR (qPT-PCR)

Total RNA was extracted from cell lines and frozen gastric adenocarcinoma tissues by the TRIzol reagent (Invitrogen). Reverse transcription of total RNA (2 Ag) was done using an Advantage RT for PCR kit (Clontech), and cDNA was subjected to PCR for 28 cycles of amplification with the following primers: CDH17 Fw, 5′-ACAATCGACCCACGTTTCTC and CHD17 Rv, 5′-ATATTGTGCACCGGGATCAT. β-Actin was used as a control.

### Immunohistochemistry (IHC)

A total of 156 formalin-fixed and paraffin-embedded gastric cancer tissue specimens were selected for IHC study. IHC staining was performed on 5-µm tissue sections rehydrated through graded alcohols. Endogenous peroxidase activity was blocked with 0.3% hydrogen peroxide for 15 min. Slides were microwave treated in 10 mM citrate buffer (pH 6.0) for 10 min. Nonspecific binding was blocked with 10% normal rabbit serum for 20 min. The tissue slides were incubated with anti-CDH17 (1∶50 dilution) for 60 min at 37°C in a moist chamber. The slides were incubated with biotinylated rabbit anti-mouse immunoglobulin at a concentration of 1∶100 for 30 min at 37°C then reacted with a streptavidin–peroxidase conjugate for 30 min at 37°C and finally incubated with 3-3′ diaminobenzidine as a chromogen substrate. To exclude a false positive signal, negative controls were included in every test by replacing the primary antibody with blocking serum. Two independent observers blinded to the clinicopathologic information scored the slides. The definition of staining was as follows: negative, with 0% to less than 5% of tumor cells showing immunoreactivity; weak, 5–40% of tumor cells showing immunoreactivity; positive, more than 40% of tumor cells showing immunoreactivity. The positive cases were futher sub-classified into CDH17+ (immunoreactivity: 40–60%), CDH17 2+ (immunoreactivity: 60–80%) and CDH17 3+ (immunoreactivity: 80–100%).

### In vitro assays to assess tumor phenotype

The experimental procedures for cell growth, foci formation, soft agar, cell migration, wound healing and cell cycle assays are described in the Supporting Methods S1.

### Rescue experiments

The rescue of CDH17 in shCDH17 AGS and MKN-45 cell lines are described in the Supporting Methods S1.

### In vivo mouse models of gastric cancer

This study was carried out in strict accordance with the recommendations in the Guide for the Care and Use of Laboratory Animals of the National Institutes of Health. The protocol was approved by the Committee on the Ethics of Animal Experiments of Sun Yat-sen University Cancer Center(Permit Number:09-0238). All surgery was performed under sodium pentobarbital anesthesia, and all efforts were made to minimize suffering. After anesthesia, Nude mice were sacrificed by cervical dislocation.

#### Tumor formation in nude mice

In vivo tumorigenesis was investigated by tumor xenograft experiments. About 2×10^6^ AGS cells infected with CDH17 shRNA, control AGS cells infected with mock RNAi vector and parental AGS cells were injected s.c. into the right and left hind legs of 4-week-old nude mice (30 mice, 10 per condition). Tumor formation in nude mice was monitored over an 8-week period. The tumor volume was calculated by the formula V = 0.5×L×W^2^. [13]

#### Tumor-bearing mouse model

Subcutaneous tumors were induced in nude mice using AGS cells as above mentioned. One week after inoculation, the mice were treated with anti-CDH17 shRNA. Each mouse received 100 µL of intratumoral injectionscontaining 1×10^9^ copies of CDH17 or mock lentivirus, twice weekly for 2 weeks. Five mice were used in each group. Tumor growth were monitored daily.

### Statistics

Statistical analyses were performed using SPSS 13.0. Differences among variables were assessed by χ2 analysis or 2-tailed Student's t tests. Data are presented as the mean ± SD unless otherwise indicated. Overall survival (OS) and disease-free survival (DFS) curves were calculated by the Kaplan-Meier method, and the differences between the two groups were compared by log-rank test. A p value of less than 0.05 was considered statistically significant.

## Results

### CDH17 is highly expressed in gastric carcinoma cells and is related to poor clinical outcome

**Figure 1:**
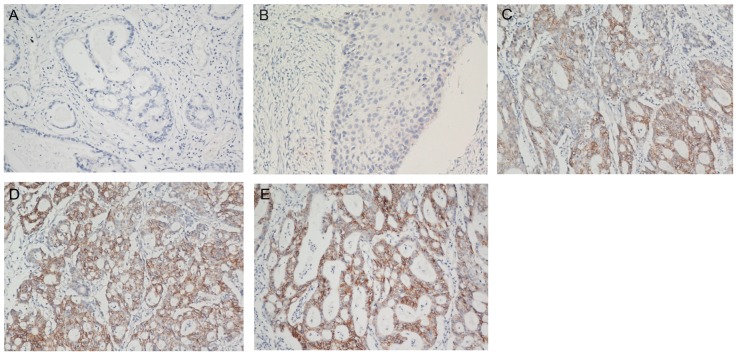
CDH17 expression in gastric carcinoma tumor samples. IHC staining in gastric carcinoma tissue (original magnification ×200). A: Negative, B: Weak, C: Positive(+), D: Positive(2+), E: Positive(3+).

First of all, we examined the expression level of CDH17 in 156 gastric carcinoma specimens by IHC: 69 (44.3%) of 156 cases were positive, whereas the remaining 87 cases (55.7%) were negative or weak for CDH17 expression (Fig. 1). The level of expression was +, 2+ and 3+ in 21 (13.5%), 20 (12.8%) and 28 (17.9%) cases respectively. These findings indicate that CDH17 may play an important role in gastric cancer tumorigenesis. We then compared the clinical and pathologic features of the patients with and without CDH17 expression. No statistical significant differences were observed between the two groups with regard to gender, age, tumor size, tumor site, depth of tumor invasion, lymph node metastasis, distant metastasis and TNM stage (Table 1).

**Table 1:** Association Between Clinicopathologic Features and CDH17 expression in 156 patients with gastric cancer.

Clinicopathological Features	CDH17 Positive (n = 69)(%)	CDH17 weak or Negative (n = 87)(%)	P value
Gender			0.18
Male	43 (62.3)	60 (69.0)	
Female	26 (37.7)	27 (21.0)	
Age			0.19
<57.2 yrs	30 (43.4)	47 (54.0)	
≥57.2 yrs	39 (56.6)	42 (46.0)	
Tumor site			0.67
Cardia	22 (25.2)	25 (36.2)	
Noncardia	47 (74.8)	63 (63.8)	
Tumor size (Mean±SD)	5.89±2.96	5.67±2.82	0.27
Histological Differentiate			0.31
Well	32 (46.4)	31 (35.6)	
Poorly	25 (36.2)	41 (47.2)	
Mucous/Signet-ring cell	12 (17.4)	15 (17.2)	
Depth of tumor invasion			0.58
T1	1 (1.4)	5 (5.8)	
T2	8 (11.6)	11 (12.6)	
T3	54 (78.3)	62 (71.3)	
T4	6 (8.7)	9 (10.3)	
Lymph-node metastasis			0.32
N0	24 (34.8)	21 (24.1)	
N1	24 (34.8)	34 (39.2)	
N2	10 (14.5)	23 (26.4)	
N3	11 (15.9)	9 (10.3)	
Distant metastases			0.74
M0	60 (87.0)	74 (85.1)	
M1	9 (13.0)	13 (14.9)	
TNM stage			0.79
I	4 (5.8)	12 (13.8)	
II	22 (31.9)	12 (13.8)	
III	24 (34.8)	41 (47.1)	
IV	19 (27.5)	22 (25.3)	

**Figure 2:**
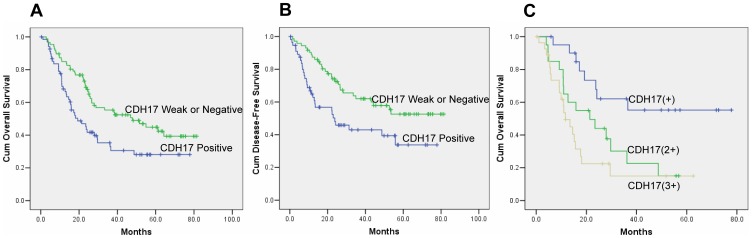
Relationship between CDH17 expression and outcome of patients with gastric cancer. Kaplan-Meier survival analysis with different CDH17 expression. A. Overall survival in 156 patients with gastric carcinoma (p<0.01) B. Disease-Free Survival in 131 gastric carcinoma patients who underwent R0 resection (p<0.01). C. Overall survival in 156 patients with gastric carcinoma according to CDH17 status (IHC 1+, 2+ and 3+)(p<0.01).

Next, we investigated the potential impact of CDH17 expression status on the long-term survival of patients by univariate and multivariate analysis. Median follow-up was 24.3 months, at the end of follow-up, 68 patients were still alive, 87 patients had died of tumor, and 1 patient had died of other cause; the cancer-related 5-year survival rate in the entire series was 37.8%. Univariate analyses showed an association between overall survival and tumor site, histological differentiation, TNM stage and CDH17 expression (Table S1). In multivariate analyses, tumor site(p = 0.02) histological differentiation (p = 0.02), TNM stage (p<0.001) and CDH17 expression (p<0.01) were independent prognostic factors for overall survival. Fig. 2A shows the overall survival difference between patients with and without CDH17 expression. Median overall survival was 15.9 months in patients with CDH17 expression compared with 26.6 months in those CDH17 negative or weak expression (P<0.01), corresponding to a 16% reduction in mortality. Furthermore, Disease-free survival(DFS) analysis was performed in 131 patients who underwent R0 resection. At the end of follow-up, 69 (52.7%) patients were still alive and 62 (47.3%) patients had died of tumor recurrence or distant metastasis, multivariate analyses showed the similar results, histological differentiation, tumor site, depth of tumor invasion (pT stage), lymph-node metastasis (pN stage) were independent prognostic factors for disease-free survival(data not shown). We also found that patients without CDH17 expression have a better prognosis than those who with CDH17 expression in tumor tissue (Fig. 2B)(p<0.01). Overall survival was reduced in patients with high expression of CDH17 (IHC 2+, 3+) than in patients with CDH17+ expression (Fig. 2C). The median overall survival of patients whose tumors had higher CDH17 expression was 10.9 months for CDH17 3+, 21.1 months for CDH17 2+, and 25.7 months in those assigned to be CDH17+ expression (p<0.01).

### CDH17 expressed in human gastric cell lines

We evaluated whether human gastric carcinoma cell lines express CDH17 and thus could be used to further evaluate the potential function this protein. We selected a panel of 6 gastric carcinoma cell lines with different metastatic potential: AGS, MKN-45, HGC-27, MGC-803 BGC-823 and SGC-7901. CDH17 mRNA level was measured by qRT-PCR analysis. Strong expression was seen in primary and metastatic gastric carcinoma cell line (AGS and MKN-45) (Fig. S1A). Western blots analysis confirmed high CDH17 protein expression in AGS (established from a primary tumor) and MKN-45 (metastatic) (Fig. S1B).

Taken together, these results suggest that CDH17 is important for initiation and metastasis of gastric cancer; at least a subset of well-credentialed gastric carcinoma cell lines retains expression of CDH17 allowing for further functional characterization.

### CDH17 knockdown induces anti-tumor effects in vitro

**Figure 3:**
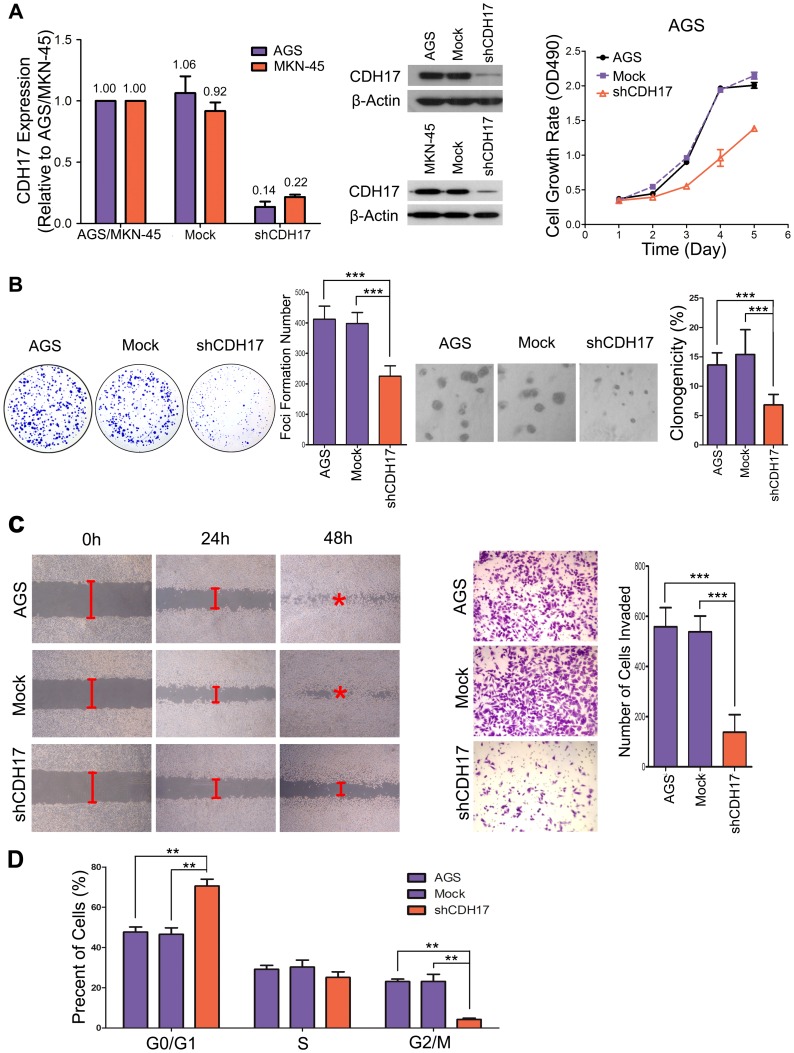
Targeting CDH17 reduced the malignant phenotype in gastric carcinoma cells. The expression of CDH17 was suppressed by lentiviral-mediated CDH17 shRNA (shCDH17); cells infected with non-targeted vector (Mock) and parental AGS or MKN-45 cells were used as controls. (A) qRT-PCR(Left) and western blot (Middle) show the mRNA and protein level of CDH17 in different experimental groups after puromycin selection. β-Actin was used as aloading control. (A) (Right) Growth curves of shCDH17 cells were compared with Mock, AGS cells by XTT assay. (Points, mean of at least three independent experiments; bars, SD). (B) Representative inhibition of foci formation in monolayer culture(Left) and colony formation in soft agar culture(Right) by CDH17 knockdown. Column chart shows quantitative analyses of foci or colonies numbers (columns, mean of at least three independent experiments; bars, SD). (C) (Left) A wound was created on a subconfluent culture of parental AGS, Mock cells and shCDH17 cells, and the rate of wound closure was monitored at 0, 24 and 48 hours. A representative photograph from three independent experiments is shown. Original magnification, ×200. (C) (Right) Representative images show the shCDH17, Mock and AGS cells that invaded through the matrigel. The number of invading tumor cells was quantified. Columns, mean of triplicate experiments; bars, SD. Original magnification, ×200. (D) Cell cycle analysis by way of flow cytometry. Knockdown CDH17 significantly impaired the tumorigenic and invasive properties of AGS cells, while inducing cell cycle G0/G1 arrest. (*P<0.05; **p<0.01; ***, P<0.001).

Next, we knocked down CDH17 in gastric carcinoma cell lines—AGS and MKN-45 using CDH17-specific shRNAs. The knockdown yielded >75% reduction in both mRNA and protein levels (Fig. 3A). We then assessed the tumorigenic and metastatic properties (proliferation, colony formation, adhesion, invasion and cell cycle) of the CDH17 deficient gastric cancer cells (shCDH17 cells) compared with the mock vector-infected control cells.

CDH17 shRNA-mediated knockdown resulted in significantly reduced cell growth of gastric carcinoma cell lines (P<0.001) (Fig. 3A, Right). Foci formation was also significantly inhibited (P<0.001) in shCDH17 cells compared with Mock cells (Fig. 3B, Left), a similar result was obtained in soft agar, in which the colony formation was significantly reduced in shCDH17 cells (P<0.001; Fig. 3B, Right). The role of CDH17 in metastasis was studied by wound-healing and transwell assays. CDH17 knockdown was able to decrease cell mobility (Fig. 3C, Left), as determined by the wound assay. Transwell assay revealed that reduced expression of CDH17 significantly decreases cell adhesion (P<0.001) (Fig. 3C, Right). To explore the mechanism underlying growth inhibition by CDH17 knockdown, the cell cycle distributions of cells with CDH17 shRNA hairpin and Mock were determined by flow cytometry: shCDH17 cells were arrested in G0/G1 phase, with fewer cells in G2/M-phase (Fig. 3D). The antitumor effect of CDH17 knockdown was also seen in the metastatic gastric carcinoma cell line MKN-45 (Fig. S2A–E). According to the accepted standards that would insure the quality and accuracy of RNAi experiments [14], we performed rescue experiments re-expressing CDH17 in AGS and MKN-45 shCDH17 cells (Fig. S3A–B). Restoration of CDH17 expression in AGS and MKN-45 cells returned the cells to the parental phenotype, confirming that the anti-tumor effects of CDH17 knockdown were not due to off target effects. The rescue experiments revealed that after restoration of CDH17 expression in those two cell lines, the tumorigenicity and metastatic potential increased to similar levels of parental AGS and MKN-45 cells (Fig. S3C–F). These results provide strong evidence of the oncogenic roles of CDH17 in gastric carcinoma cells.

### CDH17 as an In Vivo Target for Gastric Cancer Therapy

**Figure 4:**
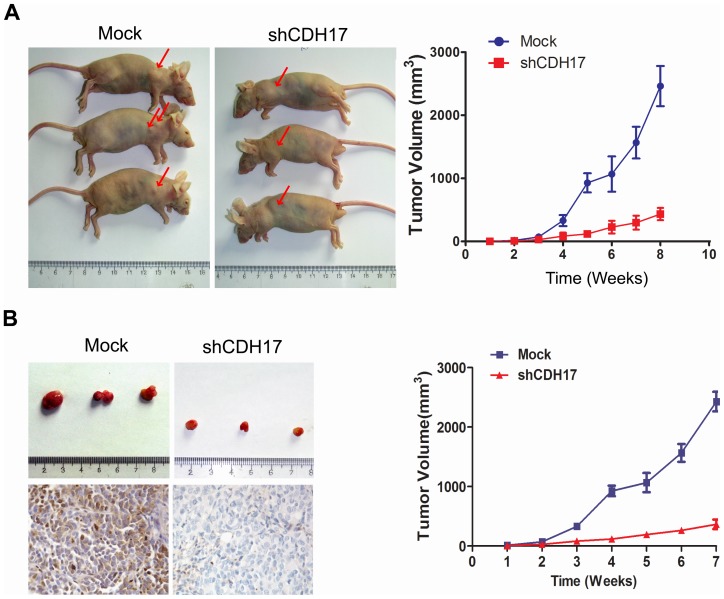
CDH17 knockdown in AGS cells reduced their tumorigenicity in vivo and were used for targeting CDH17 for gastric cancer therapy. A. Representative examples of tumors formed in nude mice following injection of shCDH17 cells in the left dorsal flanks. AGS and Mock cells were injected into the right, respectively. Line chart illustrates that the changes in volume of the subcutaneous tumors, shRNA vs. Mock,*p<0.05. B. Effect of CDH17 shRNA suppression on the growth of subcutaneous tumors in nude mice. Subcutaneous tumors were induced in nude mice by injection of AGS cells; treatment involved an intratumoral injection of Mock reagents (non-targeted RNAi vector) or shCDH17 regimen at a dose of 10^9^ viral particle/injection at the tumor site of animals (n = 3). Photographs illustrate the subcutaneous tumor produced by Mock and shCDH17 in nude mice. The volume of tumor induced in nude mice was measured among the three experimental groups after 35 days. IHC pictures demonstrate that CDH17 expression decreased in shRNA treated mice. Original magnification, ×400. Line chart illustrates that the changes in volume of the subcutaneous tumors. shRNA vs. Mock, ***p<0.001.

We examined whether CDH17 is required for tumor formation and invasiveness in gastric carcinoma cells in a nude mice model. We subcutaneously injected shCDH17, Mock and parental AGS cells into nude mice. Tumors are ordinarily visible macroscopically in the dorsal flanks within a 6 to 8-week period of cells injection. 1 mouse from both the parental AGS and Mock groups died during the third week after injection (humane endpoints were used). Mice were killed 8 weeks after tumor cell injection. In the group of mice injected with shCDH17 cells, 2 mice displayed no tumor nodule, and 8 mice showed significant reduced tumor formation compared with mice injected with mock cells. Tumor size and weight were significantly reduced in mice injected with shCDH17 cells compared with mice injected with mock cells (Fig. 4A). These results show that reduction of CDH17 expression can significantly suppress tumorigenicity in vivo.

To further evaluate the effect of CDH17 knockdown on primary tumor growth, we then investigated whether in vivo delivery of CDH17 shRNA could impede the growth of an established tumor xenograft from parental AGS cells in nude mice. One week after tumor inoculation, when the tumor had reached approximately 100 mm^3^, we performed an intratumoral injection of CDH17 shRNA or mock vector at a dose of 10^9^ viral particle/injection at the tumor site. The injection was administered twice weekly, for 2 weeks. In the animals injected with the Mock vector, the tumors grew progressively during the course of the experiment. On the contrary, intratumoral injection of CDH17 shRNA resulted in significantly smaller tumors at every time point, including the final volume measurement (Fig. 4B). Examination of the tumor cells by CDH17 IHC demonstrated remarkable down-regulation of intended CDH17 target in the shCDH17 treated cells (P<0.05; Fig. 4B). These results demonstrate that in vivo downregulation of CDH17 in gastric carcinoma cells reduces the tumor formation in mice, implying that CDH17 is a therapeutic target for gastric cancer.

### Down-regulation of Wnt/β-catenin signaling by CDH17 knockdown

**Figure 5:**
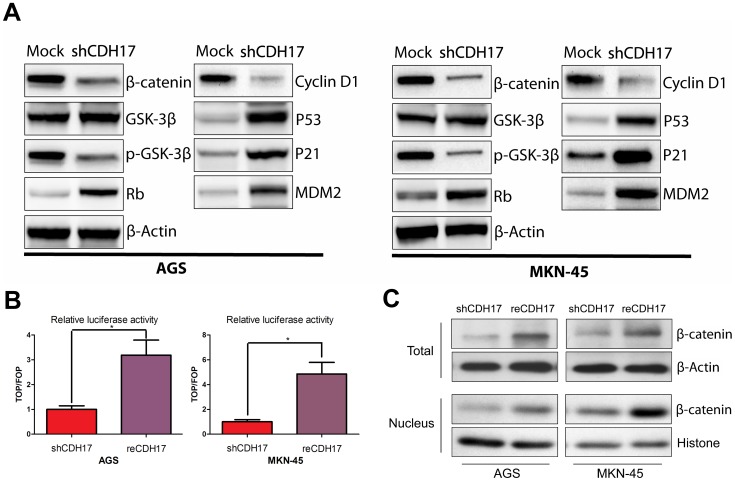
CDH17 activates multiple signal transduction pathways. A. Expression of β-catenin, GSK-3β, p- GSK-3β, Rb, Cyclin D1were compared between shCDH17 and Mock both in AGS and MKN-45 cells by Western blot analysis. MDM2, p53 and p21 also were evaluated. β-Actin was used as a loading control. B. TOPflash/FOPflash reporter assay shows that Wnt signaling re-activated after CDH17 restoration in AGS and MKN-45. (* P<0.05). C. Increase of both the total and nuclear β-catenin protein after restoration of CDH17 in AGS and MKN-45, when compared with the shCDH17.

We interrogated several pathways in order to discover the molecular mechanism of CDH17 knockdown-mediated inhibition of gastric cancer growth, proliferation and metastasis. We found several members of the Wnt/β-catenin pathway to be differentially expressed between shCDH17 and parental cells. β-catenin expression, GSK-3β phosphorylation, and Cyclin D1 expression decreased; and Rb expression increased in both AGS and MKN-45 shCDH17 cells. In addition, expression of P53, MDM2 and the key cell cycle regulator p21 increased in CDH17-knockdown cells. (Fig. 5A). In a TOPFlash/FOPFlash reporter luciferase assay (Fig. 5B), restoration of CDH17 in both AGS and MNK-45 cells significantly increased the strength of TCF/LEF signals compared with the shCDH17 controls. Furthermore, as shown by way of immunoblot analysis, we detected an increase of both the total and nuclear β-catenin protein after restoration of CDH17 in AGS and MKN-45, when compared with the shCDH17 (Fig. 5C).Together, these results indicate that CDH17 knockdown results in downregulation of the Wnt/β-catenin pathway in gastric cancer cells. This, in turn, suggests that CDH17 maintains the proliferative potential through Wnt/β-catenin pathway signaling.

## Discussion

CDH17 is a novel cadherin, its structure being different from those of the classical cadherins in that the N-terminal domain and the cytoplasmic portion displays no significant homology to classical cadherins [6]. The biologic function of CDH17 is still unknown. Numerous studies have reported elevated levels of CDH17 in various human cancers, linking expression of this protein to prognosis and risk assesment [10], [15]. CDH17 overexpression has been reported in gastric adenocarcinoma [9], hepatocellular carcinoma [16] and colorectal carcinoma [17]. This indicates that CDH17 may be crucial in the tumorigenesis of gastric and liver cancer.

In humans, CDH17 is normally expressed exclusively on the basolateral surface of hepatocytes and enterocytes. After the first report of CDH17 as an intestinal metapasia marker by Grotzinger et al [9], several investigations have evaluated CDH17 expression in gastric cancer. CDH17 was expressed in 50–78% of gastric cancer tissues with intestinal-type predominance [18], [19]. In the present study, we demonstrate that CDH17 is an oncogene and an attractive therapeutic target in gastric cancer: CDH17 is highly expressed in tumor tissues, with almost half of gastric cancers being CDH17 positive by IHC. Park and colleagues evaluated the CDH17 expression in more than 200 gastric carcinoma tissue samples, and reported that it was highly expressed in earlier TNM stages [20], Moreover, they found that reduced expression of CDH17 has been shown to be closely associated with tumor aggressiveness and lymph node metastasis of human gastric carcinoma [20]. However, others reported that its expression was much higher in advanced lymph node metastasis [18], [21]. In our study, there was no relationship between CDH17 expression and other clinicopathological features.

As a prognostic factor, CDH17 expression showed a tendency towards an indicator for unfavorable survival in multiple patient cohorts, even after controlling for tumor stage. In this context, the median overall survival of 15.9 months in the group with positive CDH17 expression, vs. 26.6 months in patients with IHC negative tumors represents a clinically significant relationship. Furthermore, an exploratory analysis showed poor overall survival in patients with high expression of CDH17 protein (IHC 2+ and 3+) as compared to patients with low expression of CDH17 protein (IHC+). These findings are noteworthy in view of the poor prognosis in this population. Collectively, CDH17 expression is a useful prognostic marker in gastric carcinoma and appears to be related to tumor progression.

The present study demonstrates that shRNA-mediated CDH17 knockdown in the highly tumorigenic gastric carcinoma cell lines AGS and MKN-45 could effectively suppress cell growth, decrease foci formation and colony formation in soft agar, as well as invasiveness and metastatic ability of gastric cancer in vitro. G1-S phase transition is a major checkpoint for cell cycle progression and p21 is one of the critical negative regulators during this transition [22]. The main cell-cycle effect of CDH17 knockdown is inhibition of G1-S phase transition through the activation of p21 was confirmed in blots studies (Fig. 5). A recent study in HCC cells found that depletion of CDH17 resulted in the inhibition of invasion, growth and metastasis in MHCC97H hepatocellular carcinoma cells [16]. CDH17 is a positive regulator for migratory, adhesive, and invasive behaviors. It is conceivable that, consistent with N-cadherin. In gastric cancer, CDH17 may mediate carcinoma cell interaction with gastric stroma and be involved in the promotion of gastric cancer metastasis by facilitating carcinoma cell migration and re-establishing homophilic cell-cell adhesion in metastasis. Our studies provide new insights into its potential role in gastric cancer initiation and progression. According to our results, CDH17 is an oncogene and an attractive therapeutic target in gastric cancer.

Accordingly, we postulate that targeting CDH17 may be used to treat gastric cancer in vivo. Mouse models of gastric cancer were used to validate the effect of targeting CDH17 as a potential treatment. Infection of the gastric carcinoma cells by CDH17 shRNA abolished their carcinogenicity in mice. In tumor-bearing mice, local delivery of lentiviral based CDH17 shRNA inhibited tumor growth, showing a striking suppression effect on tumor formation. These findings have important clinical implications for the treatment of gastric cancer. Currently, there is only one FDA–approved targeted therapy for the treatment of a subset of gastric cancer: Trastuzumab, a monoclonal antibody that interferes with the HER2 receptor. Unfortunately, HER2-expressing tumors are rare, only 22% gastric cancer patient could benefit from this therapy [23]. In contrast, CDH17 was highly expressed in gastric cancer in almost 50% of patients (Table 1).Currently, standard therapy for gastric cancer includes surgery with optimal debulking of disease followed by cytotoxic therapy. Despite these efforts, 80% of patients diagnosed with gastric cancer develop recurrent disease and only 20% of these patients survive 5 years following diagnosis [24]. Our results strongly suggest that gastric cancer patients may benefit from targeted therapy against CDH17, and warrant the exploration and design of potential CDH17 inhibitors.

The molecular mechanisms by which CDH17 regulates gastric cancer growth remain to be elucidated. A recent report showed a trans-interaction between E-cadherin and CDH17 in enterocytes during development of the intestinal epithelium [25], [26], suggesting that CDH17 might intersect with the Wnt pathway through its coordination with E-cadherin and/or associated partners. Previous studies found that targeting CDH17 inactivates Wnt/β-catenin signaling in hepatocellular carcinoma [16]. Activation of the Wnt/β-catenin signaling is found in about 30% of gastric cancer, and transgenic animal models have also demonstrated that Wnt signaling promotes gastric carcinogenesis [27]. Herein, CDH17 knockdown decreased β-catenin and GSK-3β phosphorylation, accompanied by a concomitant increase of Rb and reduction of Cyclin D1 in gastric cancer. Moreover, CDH17 knockdown in both AGS and MKN-45 cells led to cytoplasmic sequestration (or nuclear extravasation) and potentially degradation of β-catenin, which subsequently reduced TCF/LEF transactivation activity. Together, these observations point to a potential oncogenic role for CDH17 in gastric cancer though Wnt/β-catenin pathway. In addition, CDH17 knockdown increased P53 expression; a possible mechanism would be that CDH17 knockdown leads to reduced GSK-3β phosphorylation, which in turn activates p53-dependent apoptosis and antagonizes tumor growth [28]. Deficiency or loss of tumor suppressor p53 is common in various cancers, including gastric cancer, for which restoration of the TP53 gene may induce tumor apoptosis [29]. Our data show that CDH17 activate Wnt/β-catenin pathway and inactive p53 pathway, which may be particularly relevant to develop a personalized treatment strategy for those tumors with high CDH17 and deficient p53, particularly at the late advanced stages for which currently there are no effective treatments.

In conclusion, our study provides direct evidence of CDH17 as a bona fide oncogene in gastric cancer. We demonstrated the dramatic effect of CDH17 suppression in gastric cancer cells and proposed a mechanism of malignant transformation through the Wnt/β-catenin signaling pathway. We believe that CDH17 inhibition is an attractive therapeutic target in gastric cancer that should be explored in the near future. These data provide the foundation for further evaluation of CDH17 inhibition in clinical trials

## Supporting Information

Table S1 **Univariate and multivariate Cox proportional hazard model.**
(DOCX)

Figure S1 **CDH17 expression in gastric carcinoma cell lines.** qRT-PCR (A) and Western blot(B) analysis showed high CDH17 mRNA levels and protein expression in primary AGS and metastatic MKN-45 cell lines.
(TIF)

Figure S2 **Function study comparison of shCDH17, Mock and MKN-45 cells.** (A) Growth curves of shCDH17 cells were compared with Mock, MKN-45 cells by XTT assay (left). Points, mean of at least three independent experiments; bars, SD. (B) Representative inhibition of foci formation in monolayer culture by CDH17 knockdown. Column chart shows quantitative analyses of foci numbers, columns, mean of at least three independent experiments; bars, SD. (C) Inhibition of colony formation by CDH17 shRNA in soft agar culture. Chart shows quantitative analyses of colonies numbers, columns, mean of at least three independent experiments; bars, SD. (D) Representative images showed the shCDH17, Mock and MKN-45 cells that invaded through the matrigel. The number of invaded tumor cells was quantified. Columns, mean of triplicate experiments; bars, SD. (E) Cell cycle analysis by way of flow cytometry. Knockdown CDH17 significantly impaired the tumorigenic and invasive properties of MKN-45 cells, while inducing cell cycle G0/G1 arrest. (*P<0.05; **p<0.01; ***, P<0.001).
(TIF)

Figure S3 **Restoration of CDH17 in gastric cancer cells.** (A) Detection of RNA expression of CDH17 by qRT-PCR after CDH17 restoration in both AGS and MKN-45 shCDH17 cells. (B) Expression levels of CDH17 in AGS and MKN-45 cells after restoration. (C) Growth curves of AGS and MKN-45 shCDH17 cell after restoration of CDH17(reCDH17) were compared with shCDH17 cells by XTT assay (left). Points, mean of at least three independent experiments; bars, SD. (D) Representative increase of foci formation in monolayer culture by CDH17 restoration. Column chart shows quantitative analyses of foci numbers, columns, mean of at least three independent experiments; bars, SD. (E) Representative images showed the shCDH17 and reCDH17 cells that invaded through the matrigel. The number of invaded tumor cells was quantified. Columns, mean of triplicate experiments; bars, SD. (F) Cell cycle analysis by way of flow cytometry. Restoration of CDH17 significantly increased the tumorigenic and invasive properties of AGS and MKN-45 cells, while inducing less cells in G2/M phase. (*P<0.05; **p<0.01; ***, P<0.001).
(TIF)

Supporting Methods S1 (DOC)
