# Correction: Targeting CDH17 Suppresses Tumor Progression in Gastric Cancer by Downregulating Wnt/β-Catenin Signaling

**DOI:** 10.1371/journal.pone.0217124

**Published:** 2019-05-16

**Authors:** Hai-bo Qiu, Li-yi Zhang, Chao Ren, Zhao-lei Zeng, Wen-jing Wu, Hui-yan Luo, Zhi-wei Zhou, Rui-hua Xu

After publication of this article [1], concerns were raised about the following:

- In Fig 5A, β-catenin and p-GSK-3β bands in the panels of AGS and MKN-45 were duplicated.
- The tumor volume summarized in the line charts of Fig 4 do not accurately reflect the actual size of the tumor in the representative pictures shown in Fig 4.

In the raw data (S1 File), the authors found that β-catenin and p-GSK-3β were in the same film and explained that they mistakenly copied p-GSK-3β bands as β-catenin. Therefore, the authors provide a corrected Fig 5 here, according to the data obtained from the original experiments.

**Fig 5:**
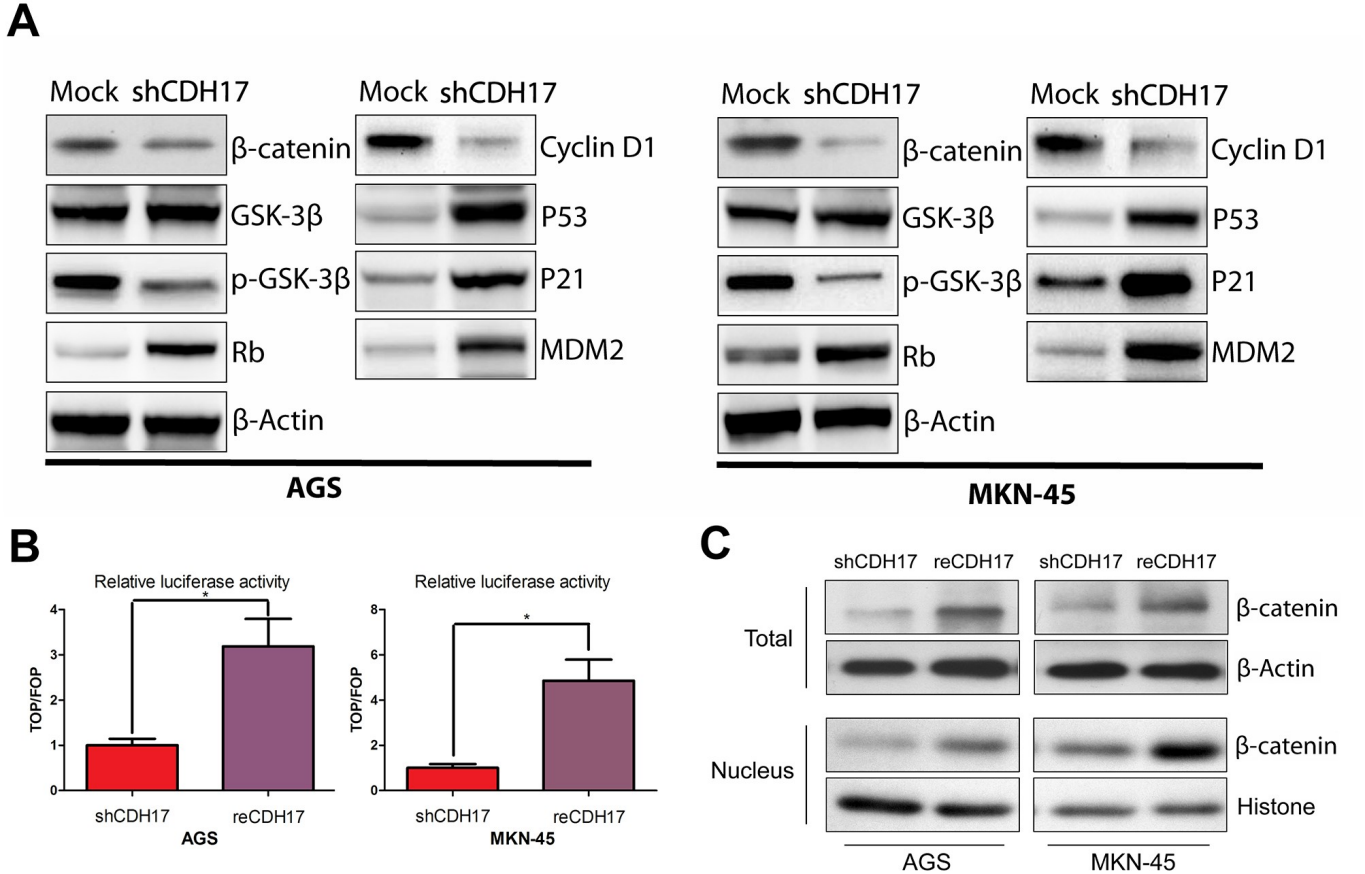
CDH17 activates multiple signal transduction pathways. A. Expression of β-catenin, GSK-3β, p-GSK-3β, Rb, Cyclin D1were compared between shCDH17 and Mock both in AGS and MKN-45 cells by Western blot analysis. MDM2, p53 and p21 also were evaluated. β-Actin was used as a loading control. B. TOPflash/FOPflash reporter assay shows that Wnt signaling re-activated after CDH17 restoration in AGS and MKN-45. (* P<0.05). C. Increase of both the total and nuclear β-catenin protein after restoration of CDH17 in AGS and MKN-45, when compared with the shCDH17.

Additional data regarding corrections to Fig 5 are provide below as S2–S5 Files.

The authors explained that when they were calculating the tumor volume in the nude mice, they wrongly used the formula Volume = L × W^2^ × 2 instead of Volume = L × W^2^/2. Due to this error, the tumor volumes summarized in the growth curve are larger than the tumor displayed in the representative pictures. The authors recalculated the data obtained from the original experiments with the correct formula (S6 File) and summarized the results in Fig 4. Please see the corrected Fig 4 here.

**Fig 4:**
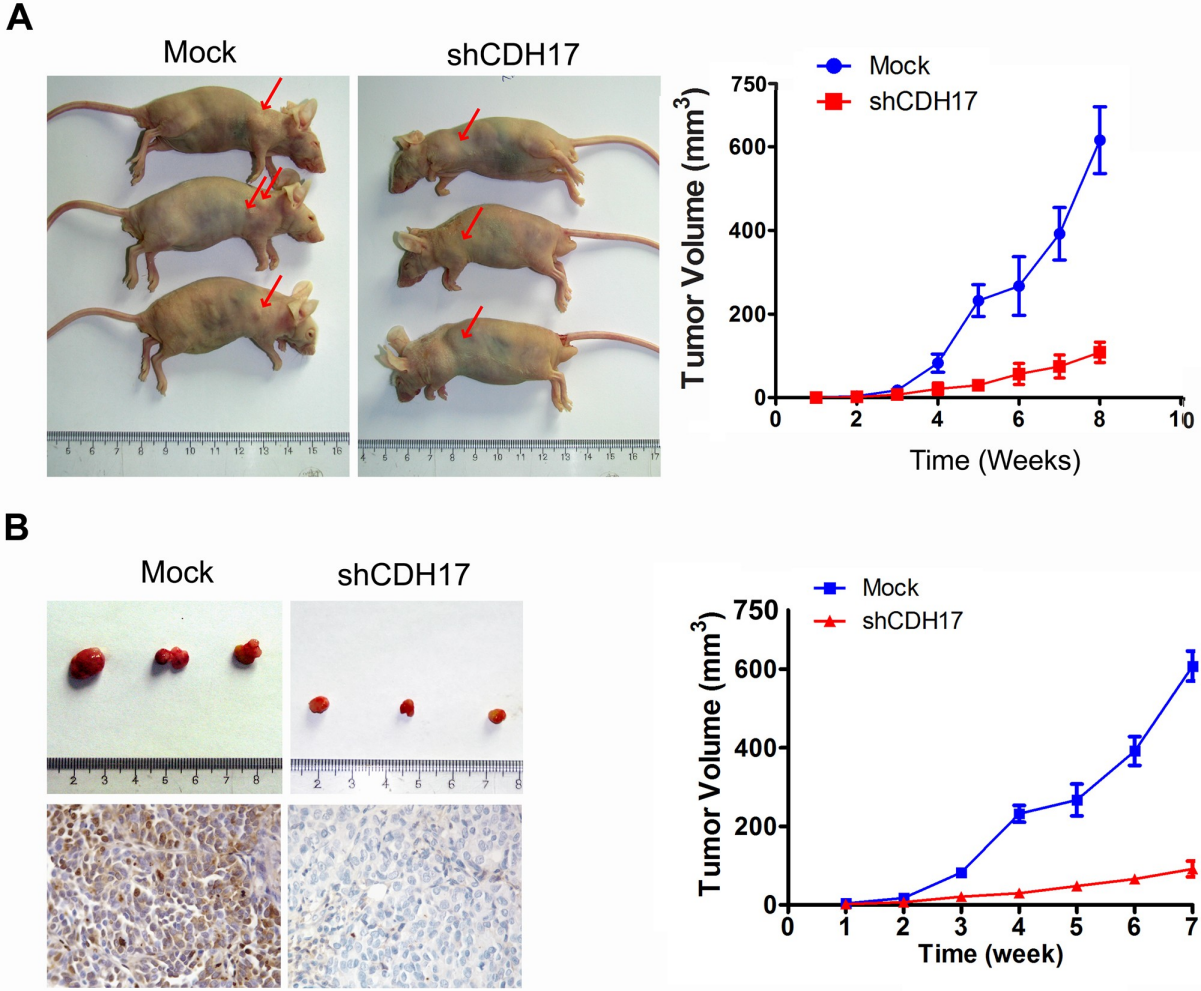
CDH17 knockdown in AGS cells reduced their tumorigenicity in vivo and were used for targeting CDH17 for gastric cancer therapy. A. Representative examples of tumors formed in nude mice following injection of shCDH17 cells in the left dorsal flanks. AGS and Mock cells were injected into the right, respectively. Line chart illustrates that the changes in volume of the subcutaneous tumors, shRNA vs. Mock,*p,0.05. B. Effect of CDH17 shRNA suppression on the growth of subcutaneous tumors in nude mice. Subcutaneous tumors were induced in nude mice by injection of AGS cells; treatment involved an intratumoral injection of Mock reagents (non-targeted RNAi vector) or shCDH17 regimen at a dose of 10^9^ viral particle/injection at the tumor site of animals (n = 3). Photographs illustrate the subcutaneous tumor produced by Mock and shCDH17 in nude mice. The volume of tumor induced in nude mice was measured among the three experimental groups after 35 days. IHC pictures demonstrate that CDH17 expression decreased in shRNA treated mice. Original magnification, ×400. Line chart illustrates that the changes in volume of the subcutaneous tumors. shRNA vs. Mock, ***p<0.001.

The authors declare that the protocol of animal experiments was approved by the Ethics Committee of Sun Yat-sen University Cancer Center. This study was carried out in strict accordance with the recommendations in the Guide for the Care and Use of Laboratory Animals of the National Institutes of Health. Gastric cancer cells were injected subcutaneously into the left and right legs of 4-week-old nude mice. Tumor growth and body weight of each mouse was monitored twice weekly over an 8-week period. Food and fluid consumption and behavioral changes were documented daily. Humane endpoint criteria were defined as when the longest axis of the tumor exceeded 1.5 cm or there was active ulceration of the tumor.

The authors declare that the raw data underlying all results reported in the article (including those for which concerns were not raised) are available. Additionally, the authors apologize for the errors in the published article.

## Supporting information

S1 File The uncropped image of β-catenin and p-GSK-3β panels in Fig 5A. A. The uncropped image of β-catenin panel. B. The uncropped image of p-GSK-3β panel. Lane 1, AGS-Mock; lane 2, AGS-shCDH17; lane 3, MKN-45-Mock; lane 4, MKN-45-shCDH17.
(TIF)

S2 File The uncropped image of β-catenin, histone and β-actin panels in Fig 5C. A. The uncropped image of β-catenin panel 2. B. The uncropped image of histone panel. C. The uncropped image of β-catenin panel 1. D. The uncropped image of β-actin panel. Lane 1, nuclear protein of AGS-shCDH17; lane 2, nuclear protein of AGS-reCDH17; lane 3, nuclear protein of MKN-45-shCDH17; lane 4, nuclear protein of MKN-45-reCDH17; lane 5, total protein of MKN-45-shCDH17; lane 6, total protein of MKN-45-reCDH17; lane 7, total protein of AGS-shCDH17; lane 8, total protein of AGS-reCDH17.
(TIF)

S3 File The generation procedure of β-catenin panel 1 in the original published Fig 5C. (DOCX)

S4 File The evidence by which the authors prove S2 File S2 is an unstitched image. (TIF)

S5 File Raw data and analytical data summarized in Fig 5B. (XLSX)

S6 File Raw data and analytical data summarized in Corrected Fig 4. (XLSX)
