# Supplementary material for: Targeting CDH17 Suppresses Tumor Progression in Gastric Cancer by Downregulating Wnt/β-Catenin Signaling
Source: PLoS One. 2013 Mar 15;8(3):e56959. doi: 10.1371/journal.pone.0056959 (PMC3598811; doi:10.1371/journal.pone.0056959)
Supplement: Table S1 — Univariate and multivariate Cox proportional hazard model. (DOCX) [file pone.0056959.s001.docx]

**Table S1:** Univariate and multivariate Cox proportional hazard model

| Variable | Univariate | | | |  | Multivariate | | | | |
| --- | --- | --- | --- | --- | --- | --- | --- | --- | --- | --- |
|  | | P Value | RR | 95%CI | | |  | P Value | RR | 95%CI |
| Tumor site | | 0.032 | 0.6 | 0.4-1.0 | | |  | 0.017 | 0.6 | 0.4-0.9 |
| Histological differentiation | | 0.025 | 1.4 | 1.0-1.8 | | |  | 0.022 | 1.4 | 1.1-1.9 |
| TNM stage | | <0.001 | 3.1 | 2.3-4.2 | | |  | <0.001 | 3.1 | 2.2-4.2 |
| CDH17 expression | | <0.01 | 1.8 | 1.2-2.8 | | |  | <0.01 | 1.9 | 1.2-2.9 |
