# Supplementary figures and images for: Targeting CDH17 Suppresses Tumor Progression in Gastric Cancer by Downregulating Wnt/β-Catenin Signaling
Source: PLoS One. 2013 Mar 15;8(3):e56959. doi: 10.1371/journal.pone.0056959 (PMC3598811; doi:10.1371/journal.pone.0056959)

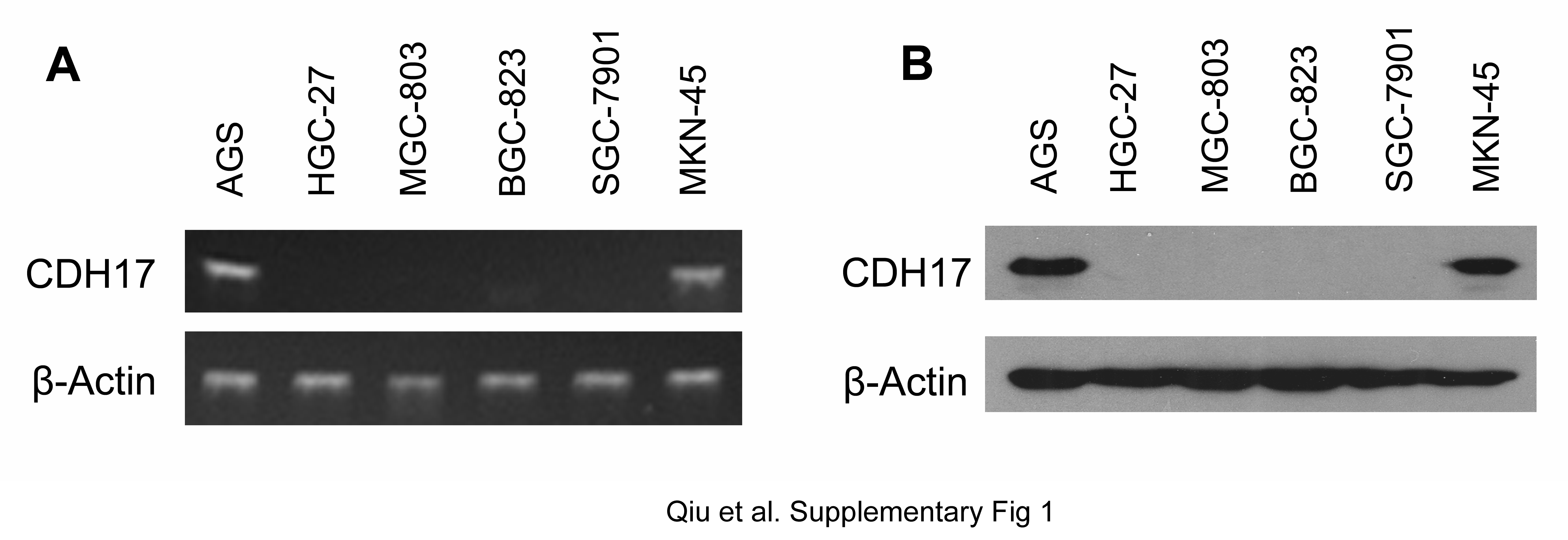

Supplement: Figure S1 — CDH17 expression in gastric carcinoma cell lines. qRT-PCR (A) and Western blot(B) analysis showed high CDH17 mRNA levels and protein expression in primary AGS and metastatic MKN-45 cell lines. (TIF) [file pone.0056959.s002.tif]

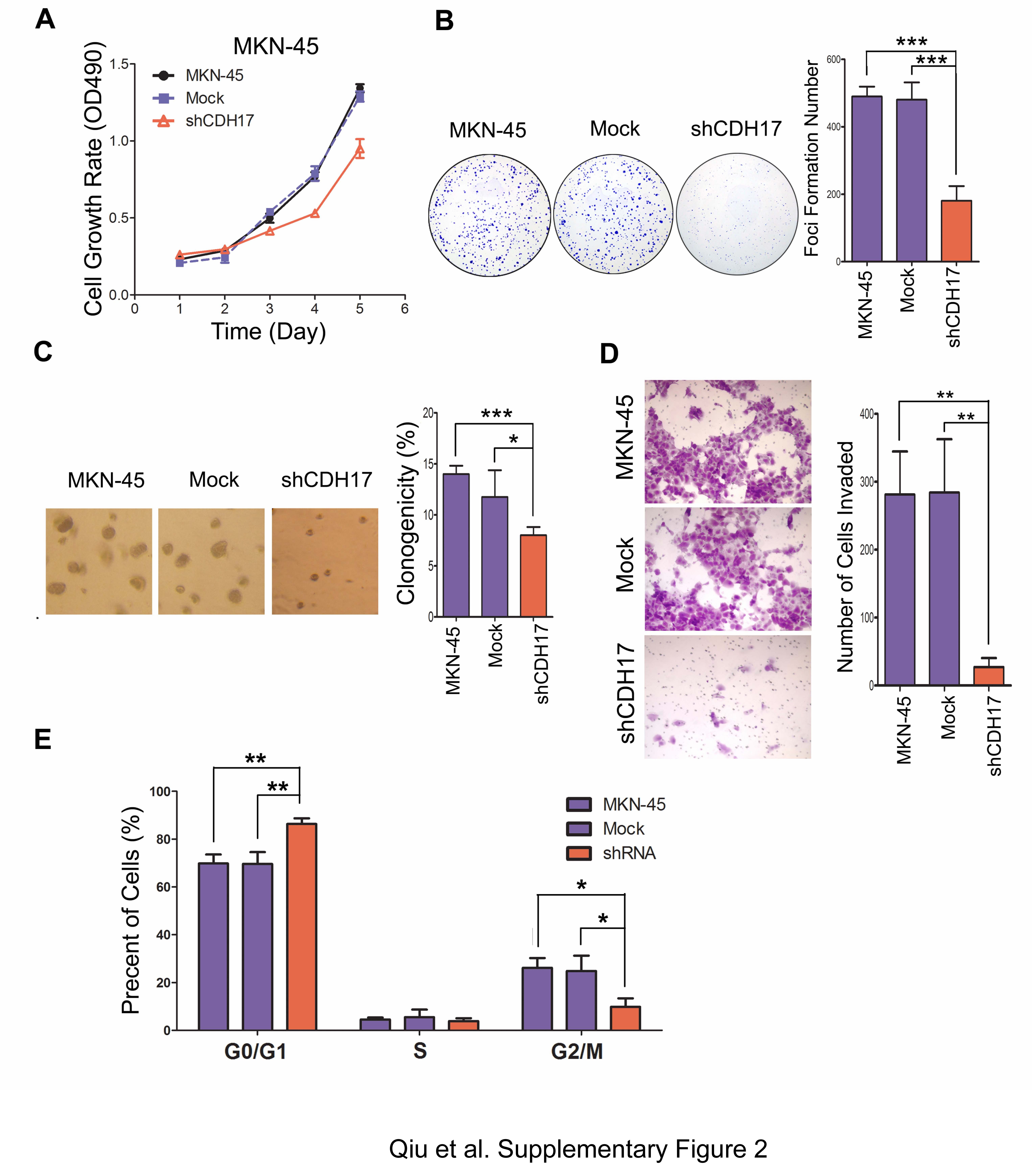

Supplement: Figure S2 — Function study comparison of shCDH17, Mock and MKN-45 cells. (A) Growth curves of shCDH17 cells were compared with Mock, MKN-45 cells by XTT assay (left). Points, mean of at least three independent experiments; bars, SD. (B) Representative inhibition of foci formation in monolayer culture by CDH17 knockdown. Column chart shows quantitative analyses of foci numbers, columns, mean of at least three independent experiments; bars, SD. (C) Inhibition of colony formation by CDH17 shRNA in soft agar culture. Chart shows quantitative analyses of colonies numbers, columns, mean of at least three independent experiments; bars, SD. (D) Representative images showed the shCDH17, Mock and MKN-45 cells that invaded through the matrigel. The number of invaded tumor cells was quantified. Columns, mean of triplicate experiments; bars, SD. (E) Cell cycle analysis by way of flow cytometry. Knockdown CDH17 significantly impaired the tumorigenic and invasive properties of MKN-45 cells, while inducing cell cycle G0/G1 arrest. (*P<0.05; **p<0.01; ***, P<0.001). (TIF) [file pone.0056959.s003.tif]

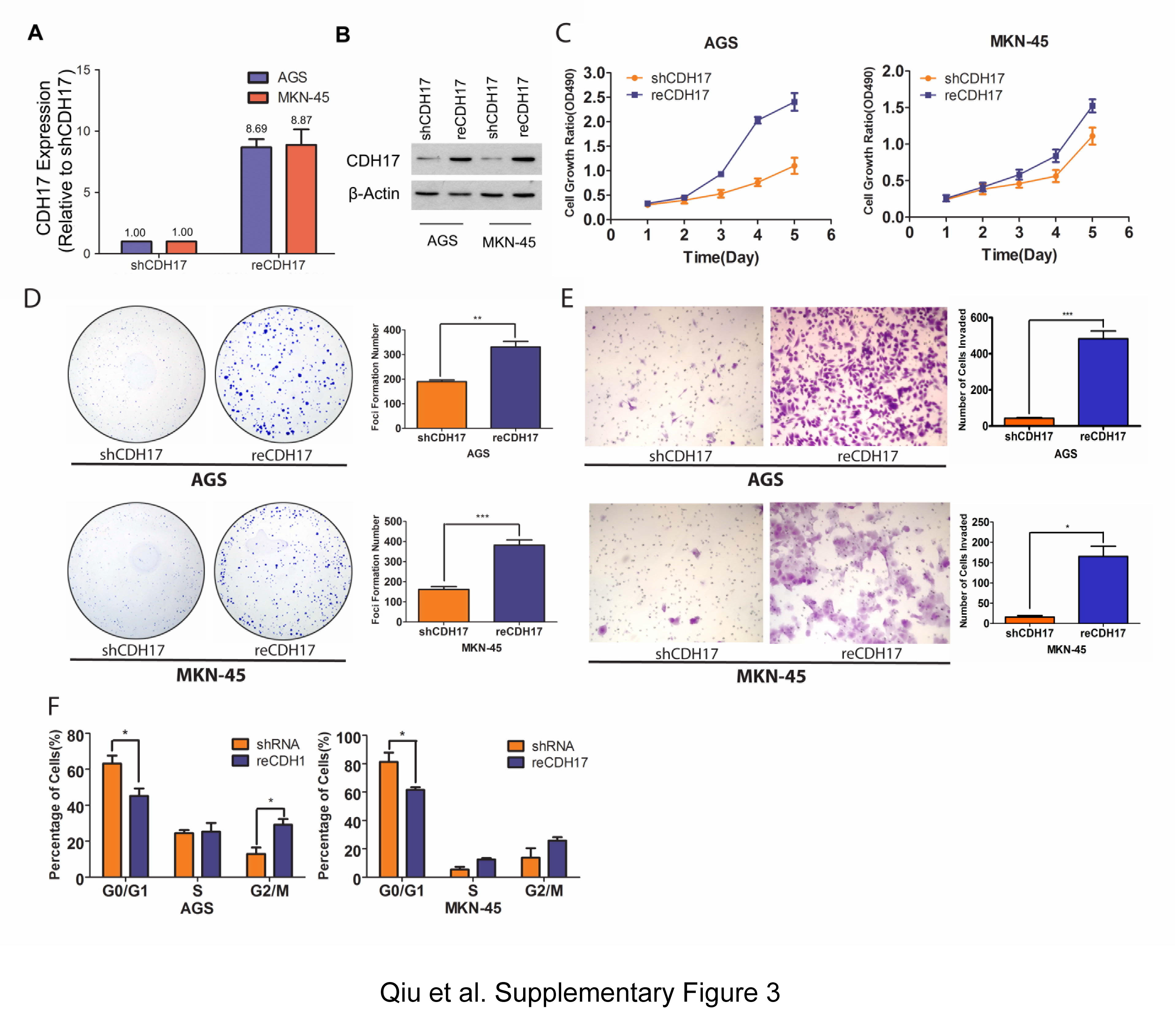

Supplement: Figure S3 — Restoration of CDH17 in gastric cancer cells. (A) Detection of RNA expression of CDH17 by qRT-PCR after CDH17 restoration in both AGS and MKN-45 shCDH17 cells. (B) Expression levels of CDH17 in AGS and MKN-45 cells after restoration. (C) Growth curves of AGS and MKN-45 shCDH17 cell after restoration of CDH17(reCDH17) were compared with shCDH17 cells by XTT assay (left). Points, mean of at least three independent experiments; bars, SD. (D) Representative increase of foci formation in monolayer culture by CDH17 restoration. Column chart shows quantitative analyses of foci numbers, columns, mean of at least three independent experiments; bars, SD. (E) Representative images showed the shCDH17 and reCDH17 cells that invaded through the matrigel. The number of invaded tumor cells was quantified. Columns, mean of triplicate experiments; bars, SD. (F) Cell cycle analysis by way of flow cytometry. Restoration of CDH17 significantly increased the tumorigenic and invasive properties of AGS and MKN-45 cells, while inducing less cells in G2/M phase. (*P<0.05; **p<0.01; ***, P<0.001). (TIF) [file pone.0056959.s004.tif]
