# Supplementary material for: Targeting CDH17 Suppresses Tumor Progression in Gastric Cancer by Downregulating Wnt/β-Catenin Signaling
Source: PLoS One. 2013 Mar 15;8(3):e56959. doi: 10.1371/journal.pone.0056959 (PMC3598811; doi:10.1371/journal.pone.0056959)
Supplement: Supporting Methods S1 — (DOC) [file pone.0056959.s005.doc]

**Supplementary Methods**

**In vitro assays to assess tumor phenotypes**

**Cell growth assay.** Cell growth rates of cells were detected by XTT assay. Briefly, cells were seeded onto 96-well plate at a density of 1×10^4^ cells per well and incubated in 100µl DMEM with or without serum for 1 to 5 d. The cell growth rate was detected using cell proliferation XTT kit (Roche) according to manufacturer’s instructions. Triplicate independent experiments were done.

**Foci formation assay**. 1×10^3^ cells were plated in wells of a six-well plate. After 7 days culture, surviving colonies (>50 cells per colony) were counted with Giemsa staining. Triplicate independent experiments were done.

**Soft agar assay.** Anchorage-independent growth assay in cells was carried out by growing 1×10^4^ cells in 0.4% bactoagar on a bottom layer of solidified 0.6% bactoagar in 6-well plates. After 2 weeks, colonies formed in the top layer were counted and colony formation rate was calculated as percentage of total seeded cells. Triplicate independent experiments were done.

**Migration assay.** Cells were serum starved for 24 h, then 2×10^4^ cells were plated into the upper chamber of a polycarbonate transwell filter chamber coated with Matrigel (BD) and incubated for 22 hours. Cells inside the chamber were removed with cotton swabs and migratory cells on the lower membrane surface were fixed in 1% paraformaldehyde, stained with Trypanblue and counted (10 random ×100 fields per well). Cell counts are expressed as the mean number of cells per field of view. Three independent experiments were performed.

**Wound healing assay.** Cells were seeded in 6-well plates and grown under permissive conditions until reaching 90% confluence. The cells were then serum starved for 24h, and a linear wound was created in the confluent monolayer using a pipette tip. Wounds were observed and photographed at various times as indicated in the figure legends. Wound size was measured randomly at five sites perpendicular to the wound. Each experiment was repeated at least three times.

**Cell cycle analysis**. 1×10^6^ cells were cultured in RPMI medium containing 10% fetal bovine serum (FBS). Serum was withdrawn from culture medium when cells were 70% confluent. After 72 h, 10% FBS was added in the medium for an additional 12 h. Cells were fixed in 70% ethanol, stained with propidium iodide, and DNA content was analyzed by Cytomics FC (Beckman Coulter).

**Construction of CDH17 re-expression vector**

The coding sequence for CDH17 was amplified by PCR using proofreading enzymes and a DNA template obtained from the IMAGE clone reference 3451538 (Geneservice, Source Bioscience LifeSciences, Nottingham, UK). Primer sequences were: CDH17fp 5′- TGTACAAAAAAGTTGGCACC-3′ and CDH17rp 5′- TTGCCAACTTTCTTGTACAAAGT -3′. The PCR product was cloned into the pCR 2.1 vector using the TOPO TA cloning kit (Invitrogen) following the manufacturer's instructions. The CDH17 coding sequence was then excised with EcoRI and XhoI digestion and shuttled into the similarly digested expression vector pCMVtag2 (Stratagene, Agilent Technologies, Santa Clara, California, USA) to produce the expression construct pCMV-MLH1. The sequence, orientation and reading frame were confirmed by bidirectional sequencing as previously described. For forced expression studies, approximately 5×10^5^ cells per well were seeded in a six-well plate (Costar, Sigma-Aldrich, Poole, UK) and transfected with 4 μg of the expression construct using lipofectamine 2000 (Invitrogen) in accordance with the manufacturer's instructions. These are henceforth annotated as AGS reCDH17 and MKN-45 reCDH17.

**TOP/FOP-luciferase reporter assay**

AGS and MKN-45 cells were cultured to confluency, after which they were transfected with a plasmid carrying a luciferase reporter system under the TOP/FOP promoter using FuGene HD transfection reagent (from Roche Diagnostics, Mannheim, Germany) in serum-free media (Opti-MEM; Gibco, Grand Island, NY) according to the manufacturer's instructions. After 12 h, cells were washed, fresh serum-reduced media (1%) was added and cells were subjected to standard multiple scrape-wounding. The plasmid containing a TCF/LEF-dependently expressed luciferase reporter. Cells were lysed using cell culture lysis reagent (from Promega, Madison, WI, USA) 13 hours and 24 hours after wounding, respectively, and luciferase activity was determined in cell lysates using a Luciferase Assay System (from Promega, Madison, WI, USA) as described by the manufacturer. Light emission readings were performed using the Varioskan Flash Reader (from Thermo Scientific, Waltham, MA) according to the manufacturer's instructions. Values were normalized to total protein content in cell lysates generated from similarly treated wells (mean values of 3 wells) as determined using the BCA Protein Assay (from Thermo Scientific, Waltham, MA).
