# Supplementary figures and images for: Correction: Targeting CDH17 Suppresses Tumor Progression in Gastric Cancer by Downregulating Wnt/β-Catenin Signaling
Source: PLoS One. 2019 May 16;14(5):e0217124. doi: 10.1371/journal.pone.0217124 (PMC6521985; doi:10.1371/journal.pone.0217124)

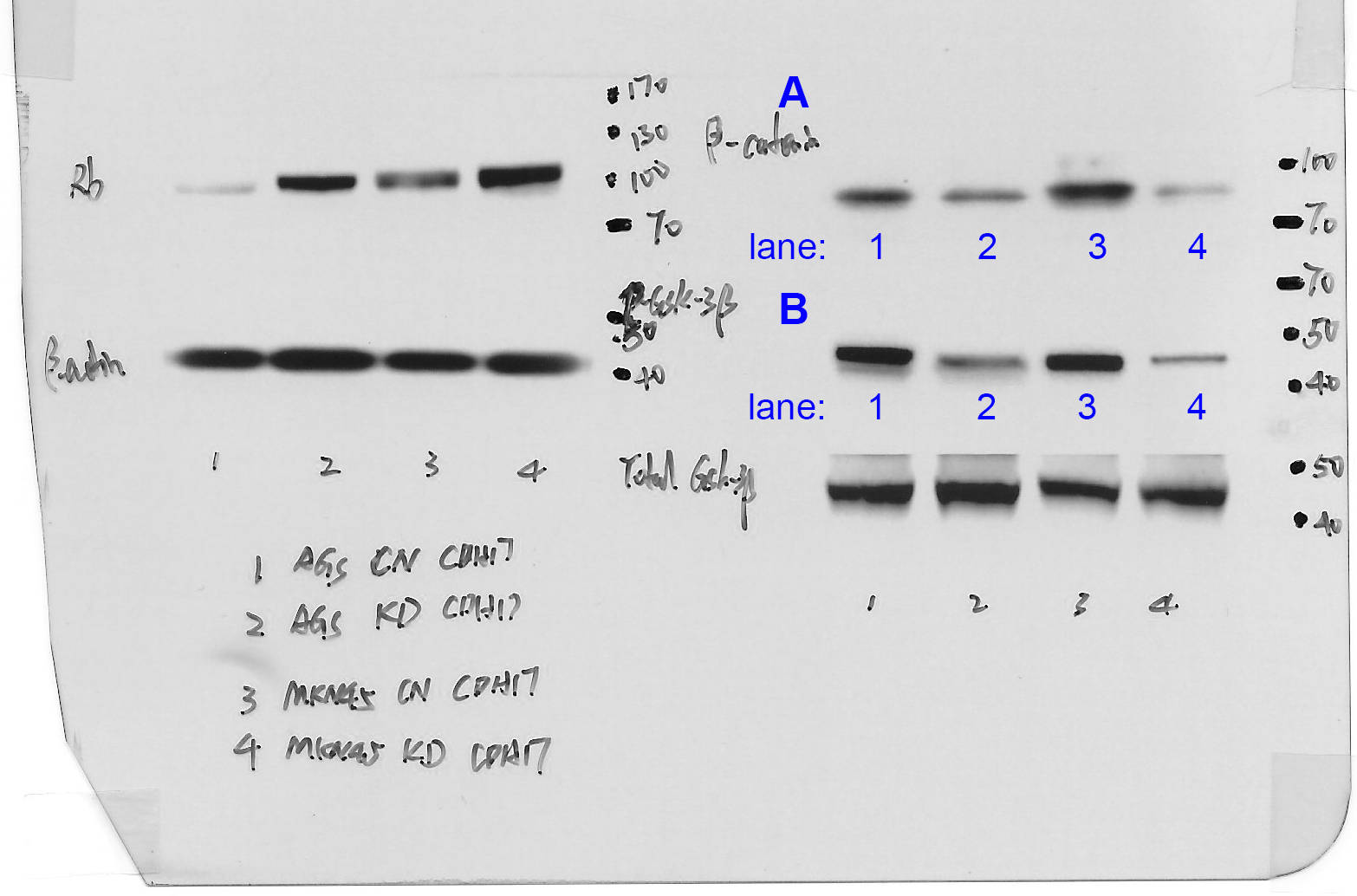

Supplement: S1 File — A. The uncropped image of β-catenin panel. B. The uncropped image of p-GSK-3β panel. Lane 1, AGS-Mock; lane 2, AGS-shCDH17; lane 3, MKN-45-Mock; lane 4, MKN-45-shCDH17. (TIF) [file pone.0217124.s001.tif]

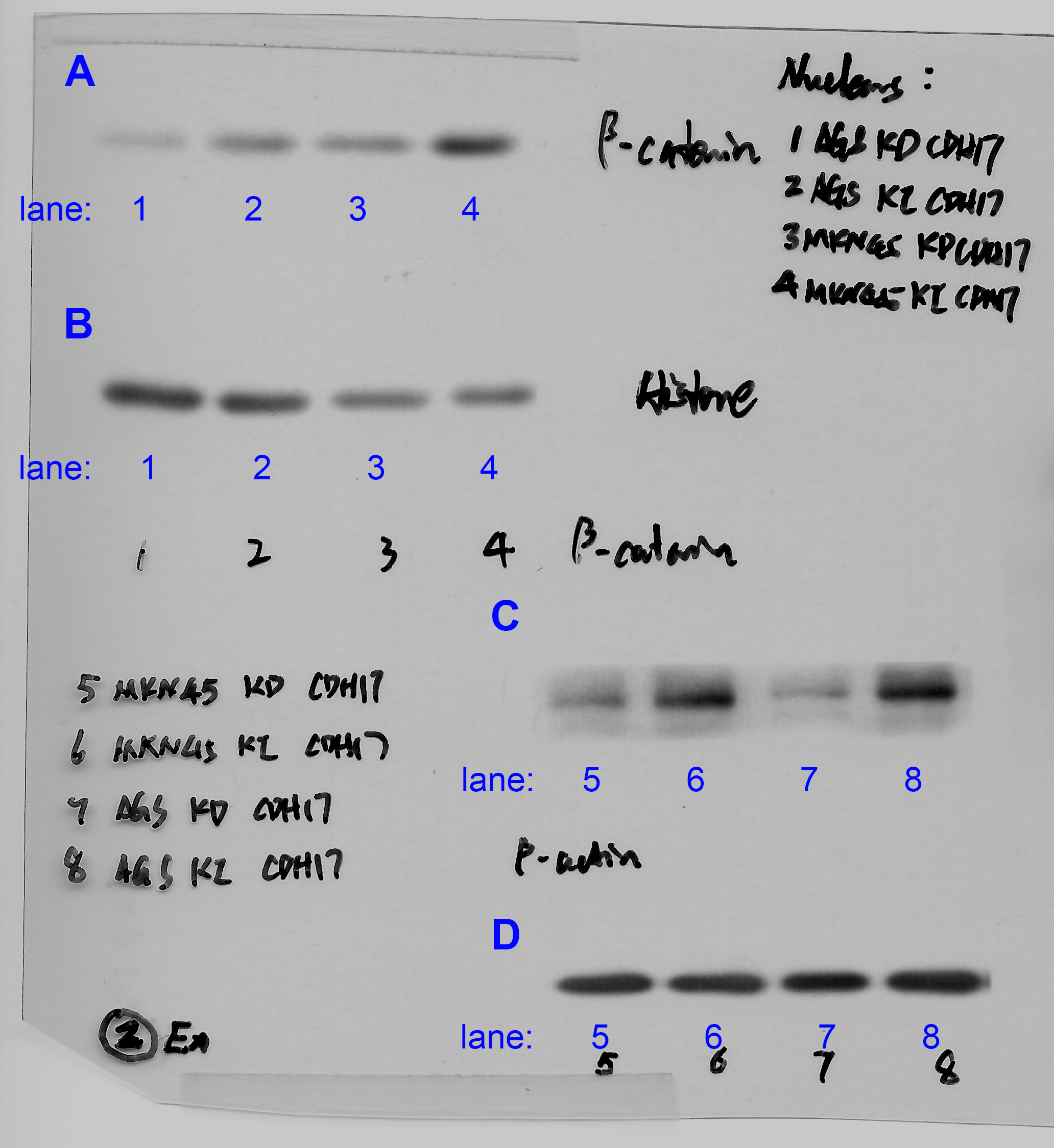

Supplement: S2 File — A. The uncropped image of β-catenin panel 2. B. The uncropped image of histone panel. C. The uncropped image of β-catenin panel 1. D. The uncropped image of β-actin panel. Lane 1, nuclear protein of AGS-shCDH17; lane 2, nuclear protein of AGS-reCDH17; lane 3, nuclear protein of MKN-45-shCDH17; lane 4, nuclear protein of MKN-45-reCDH17; lane 5, total protein of MKN-45-shCDH17; lane 6, total protein of MKN-45-reCDH17; lane 7, total protein of AGS-shCDH17; lane 8, total protein of AGS-reCDH17. (TIF) [file pone.0217124.s002.tif]

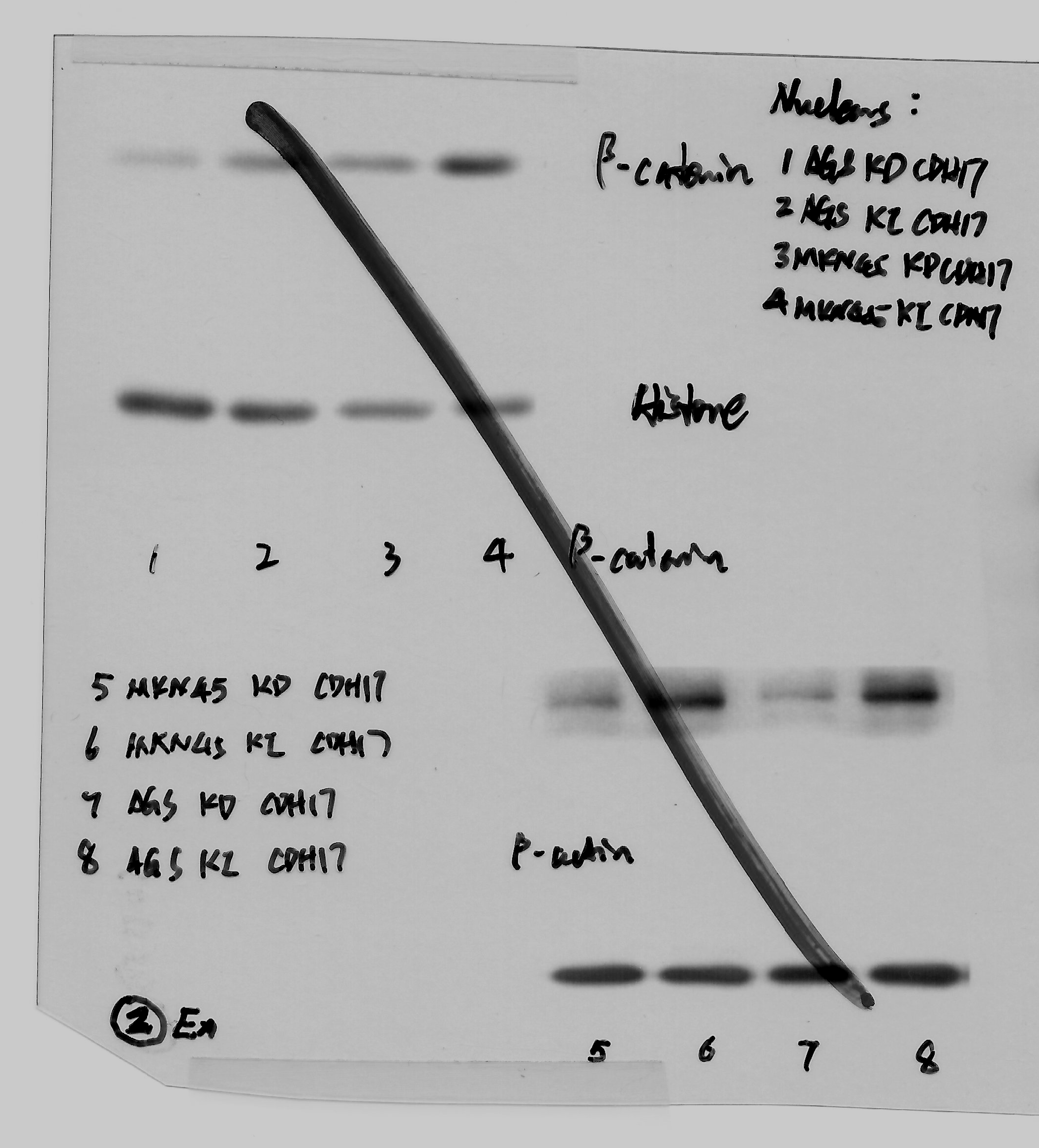

Supplement: S4 File — (TIF) [file pone.0217124.s004.tif]
