# Supplementary material for: Correction: Targeting CDH17 Suppresses Tumor Progression in Gastric Cancer by Downregulating Wnt/β-Catenin Signaling
Source: PLoS One. 2019 May 16;14(5):e0217124. doi: 10.1371/journal.pone.0217124 (PMC6521985; doi:10.1371/journal.pone.0217124)

**File S3.** The generation procedure of β-catenin panel 1 in the original published Figure 5C.

1. Open the original published Figure 5 and raw data in Photoshop.


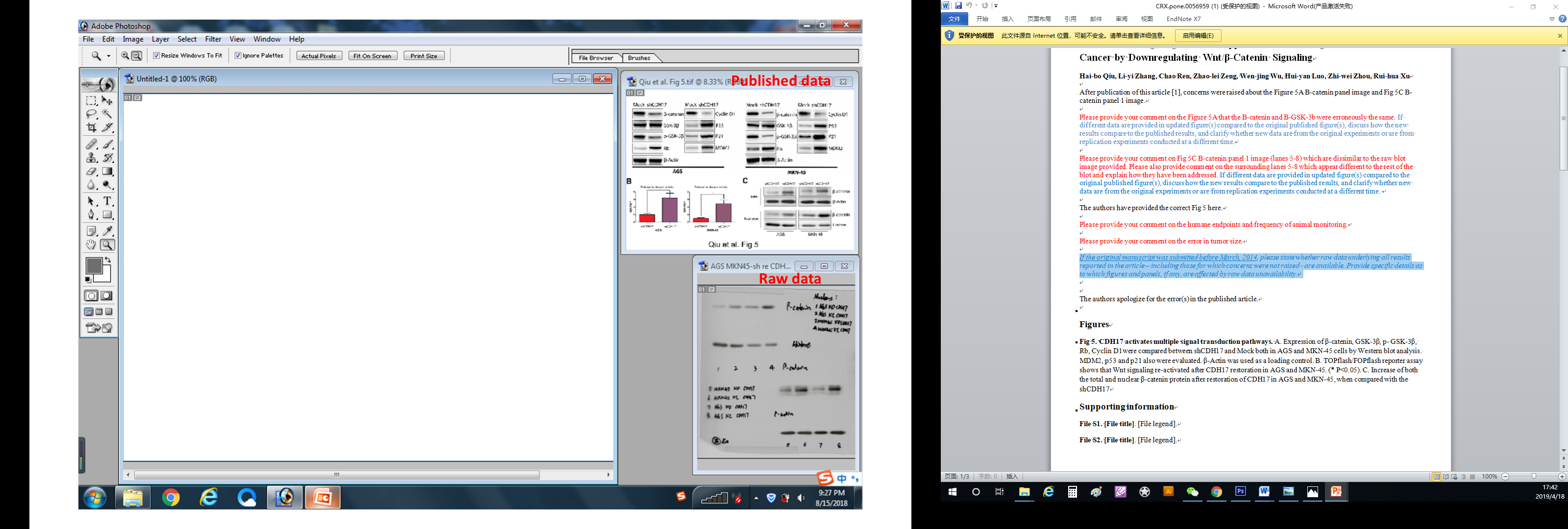


2. Copy the β-catenin panel 1 in the published data and paste it into a new file.


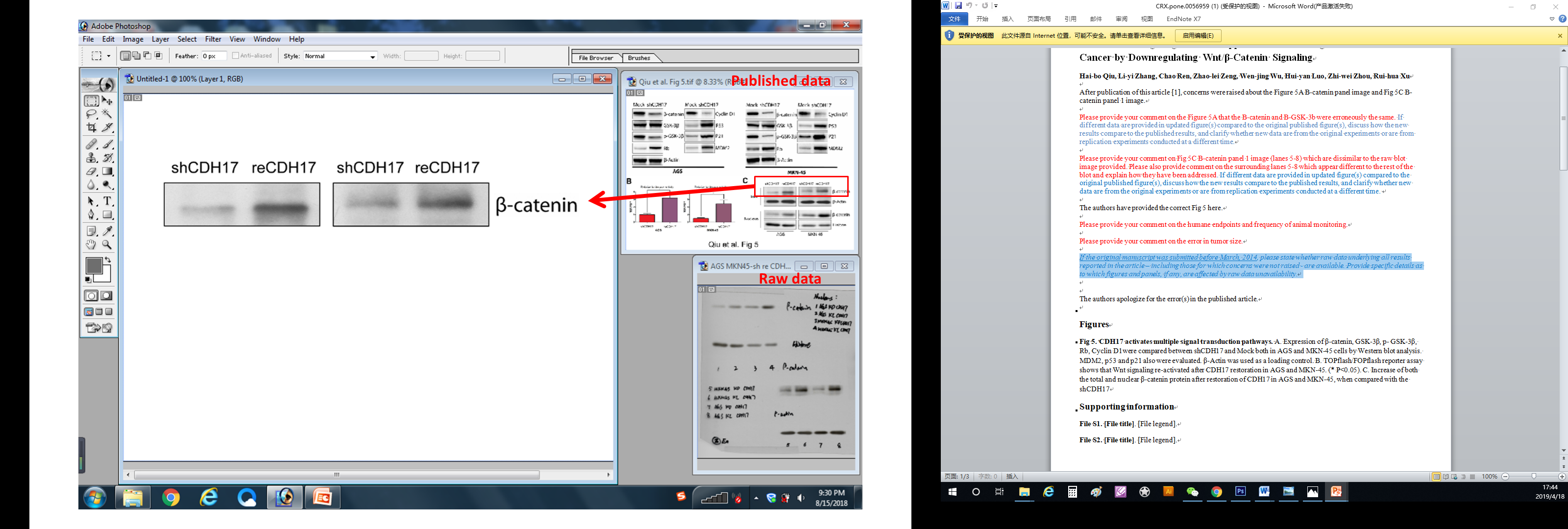


3. Copy the β-catenin panel 1in the raw data and paste it in the same file.


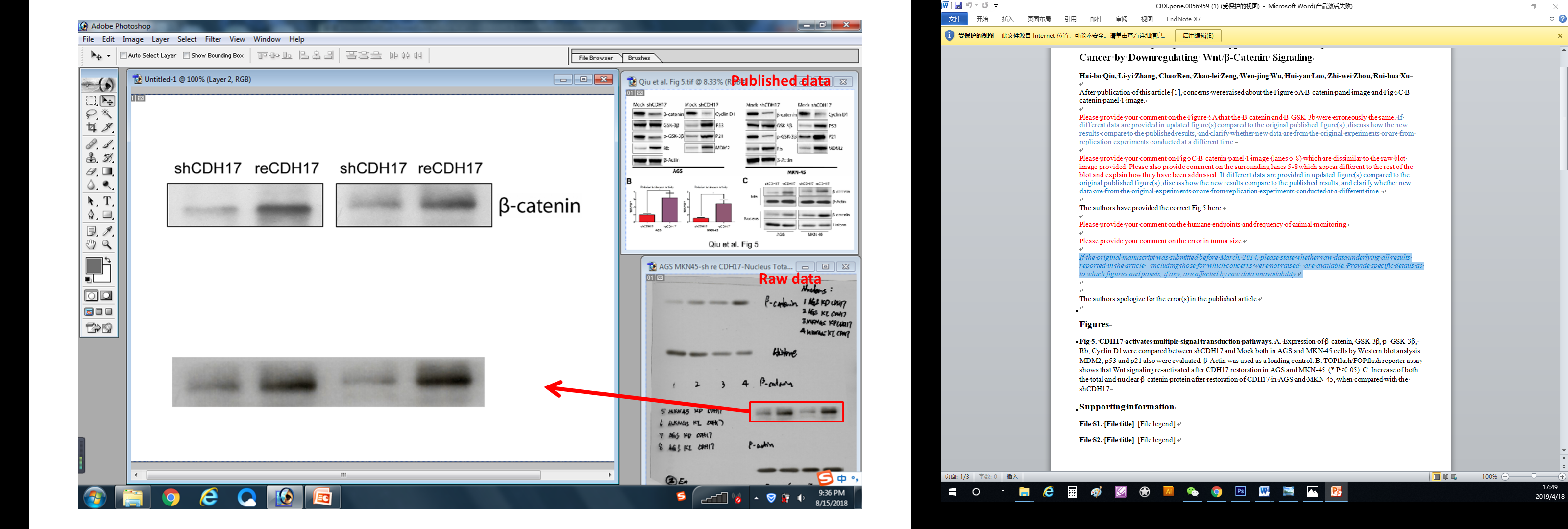


4. Copy and paste the lane 7 and 8.


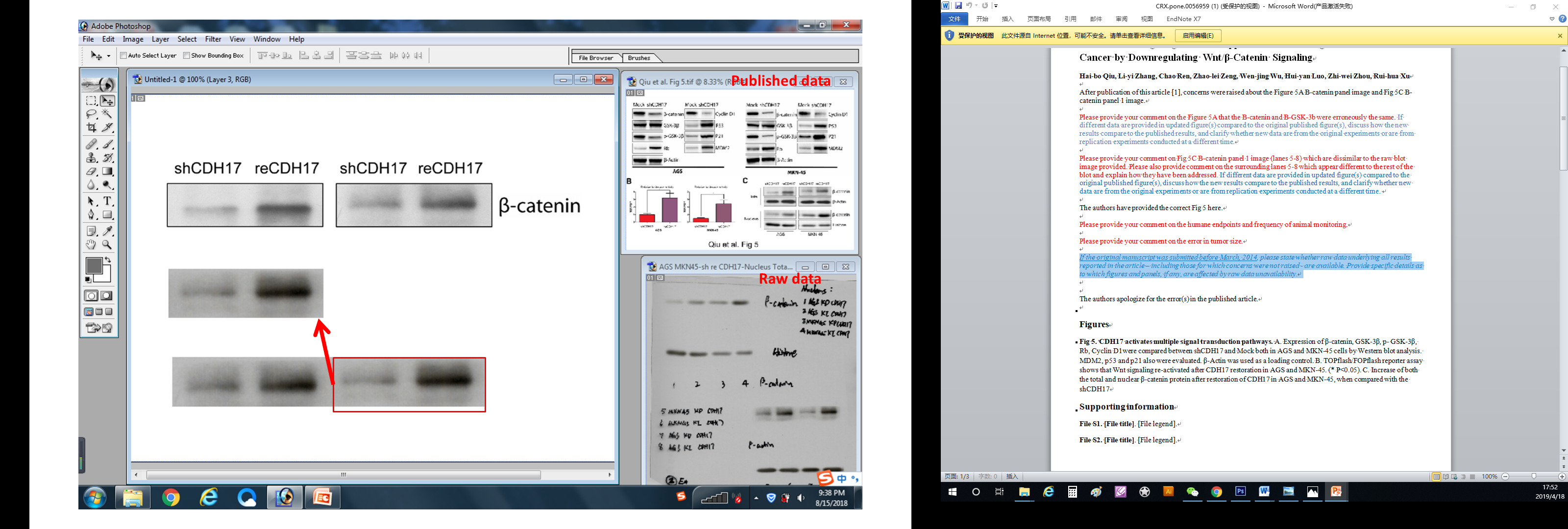


5. Transform the lane 7 and 8 (flip vertical).


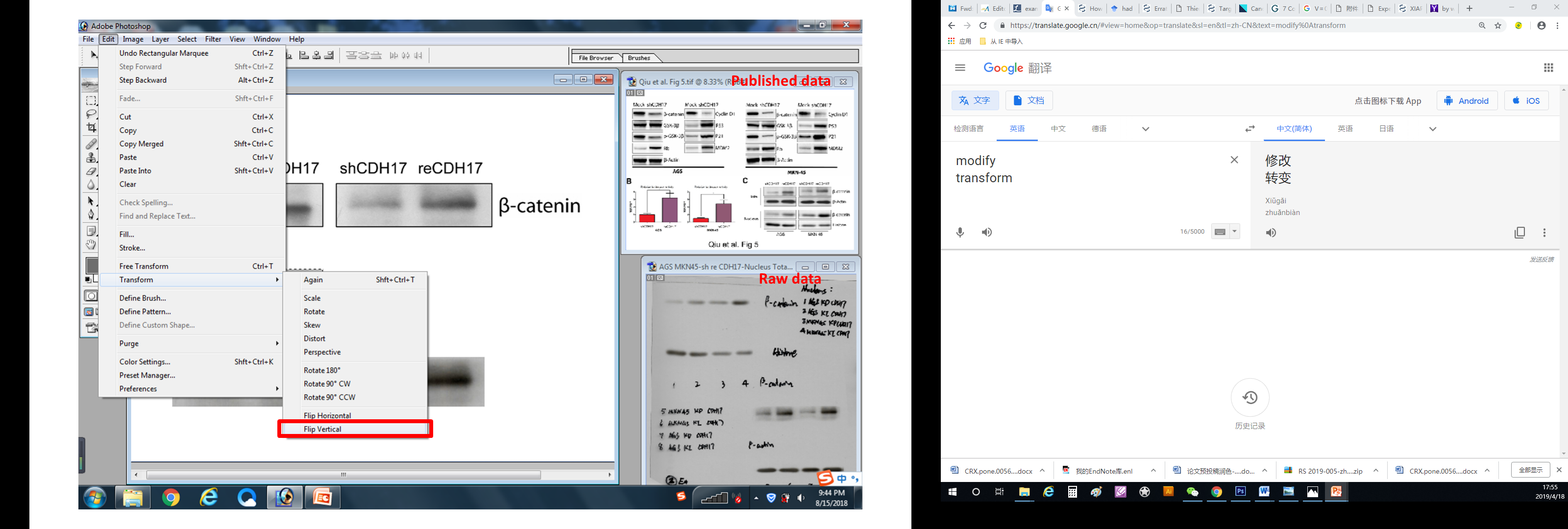


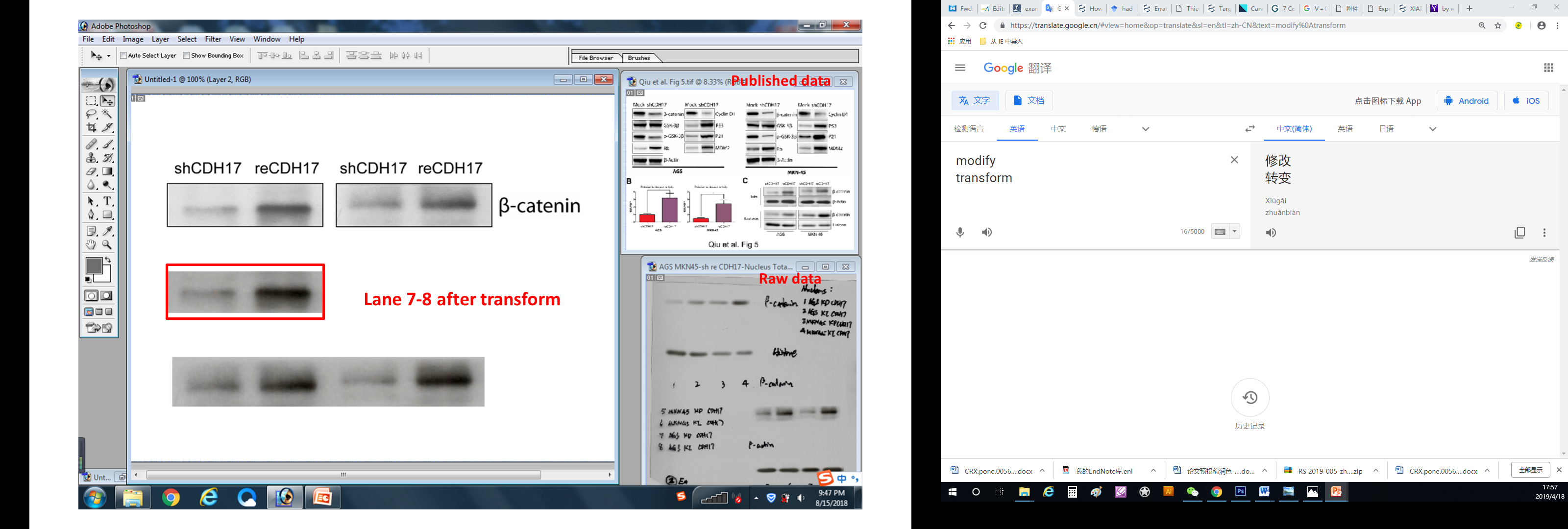


6. Adjust the brightness/contrast of the lane 7 and 8.


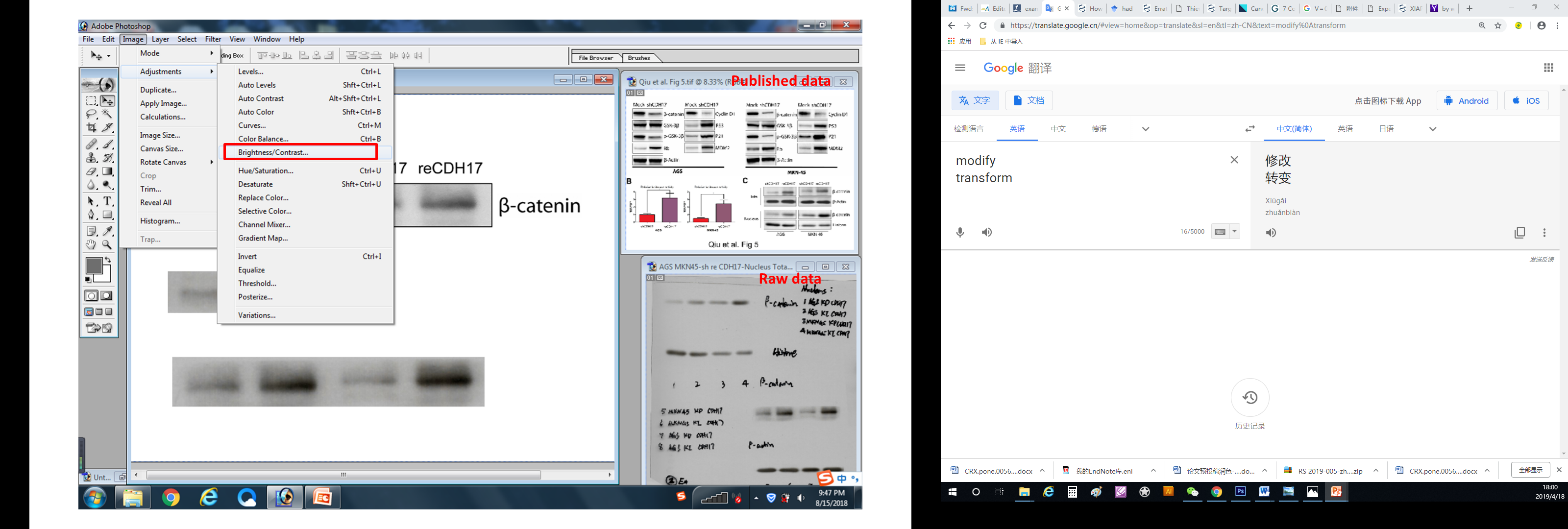


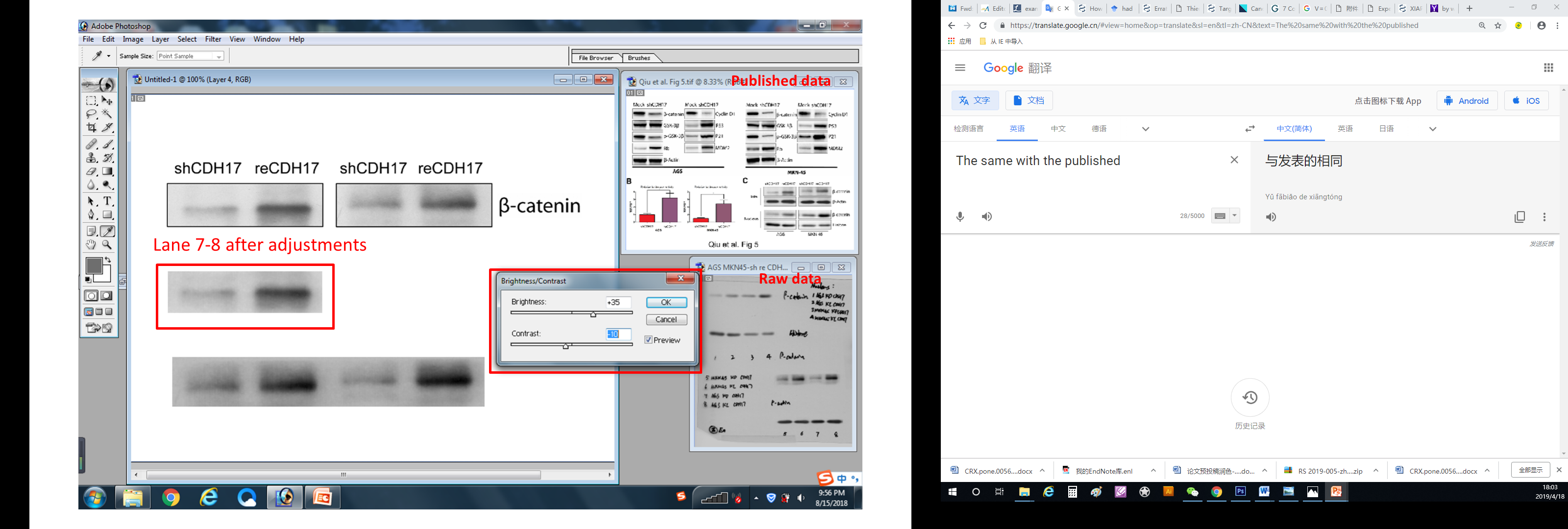


7. Copy and paste the lane 5 and 6.


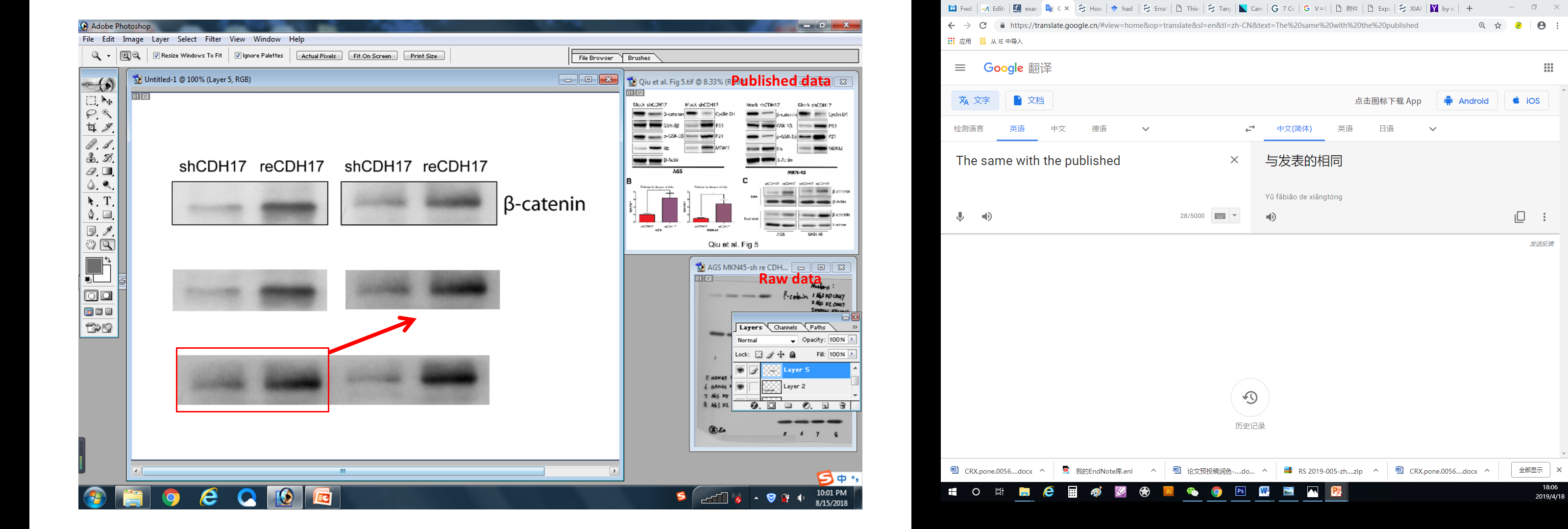


8. Adjust the brightness/contrast of the lane 5 and 6.


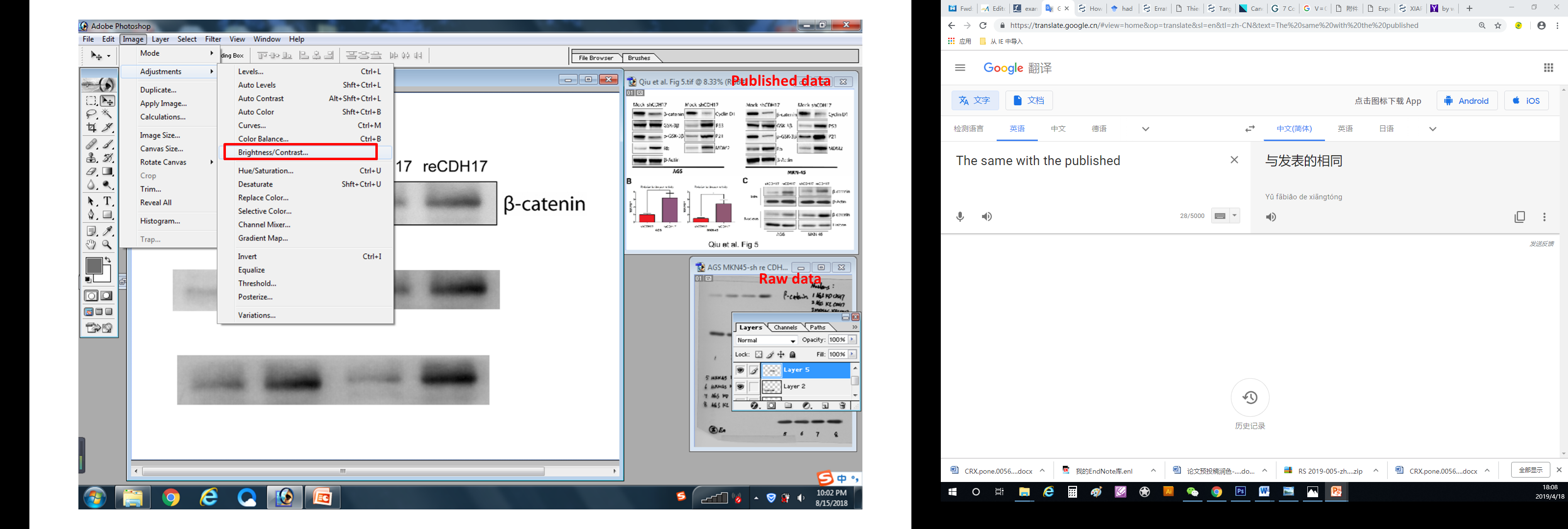


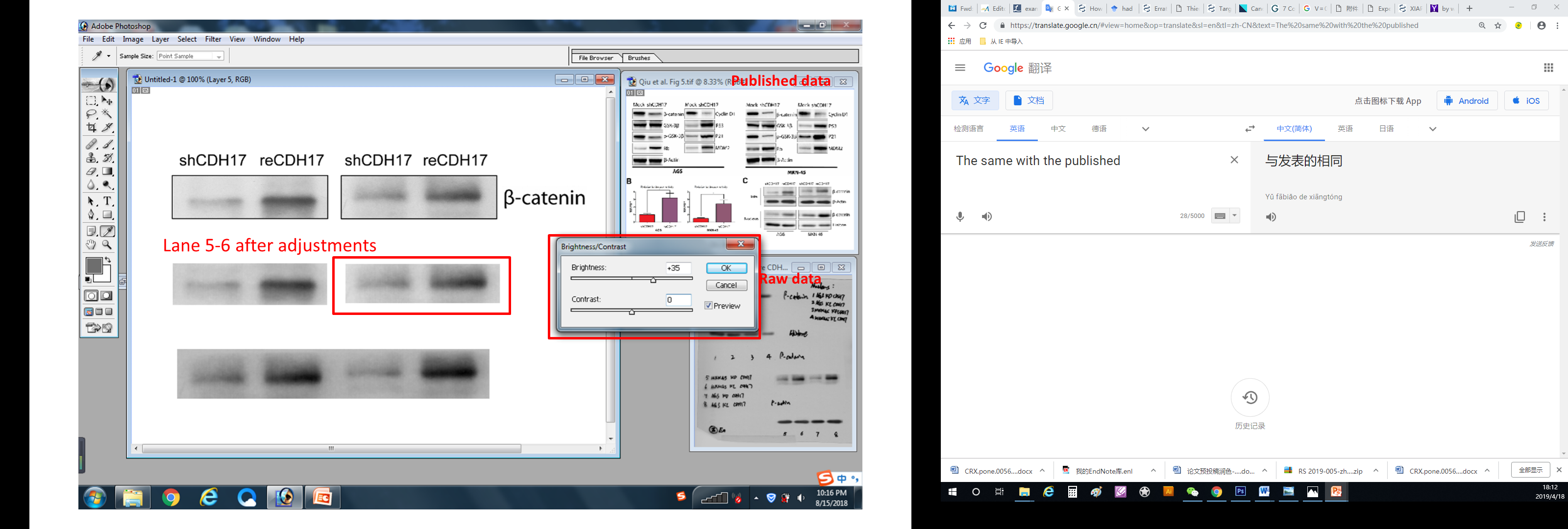


9. The published image was the same with the image generated from the raw data.


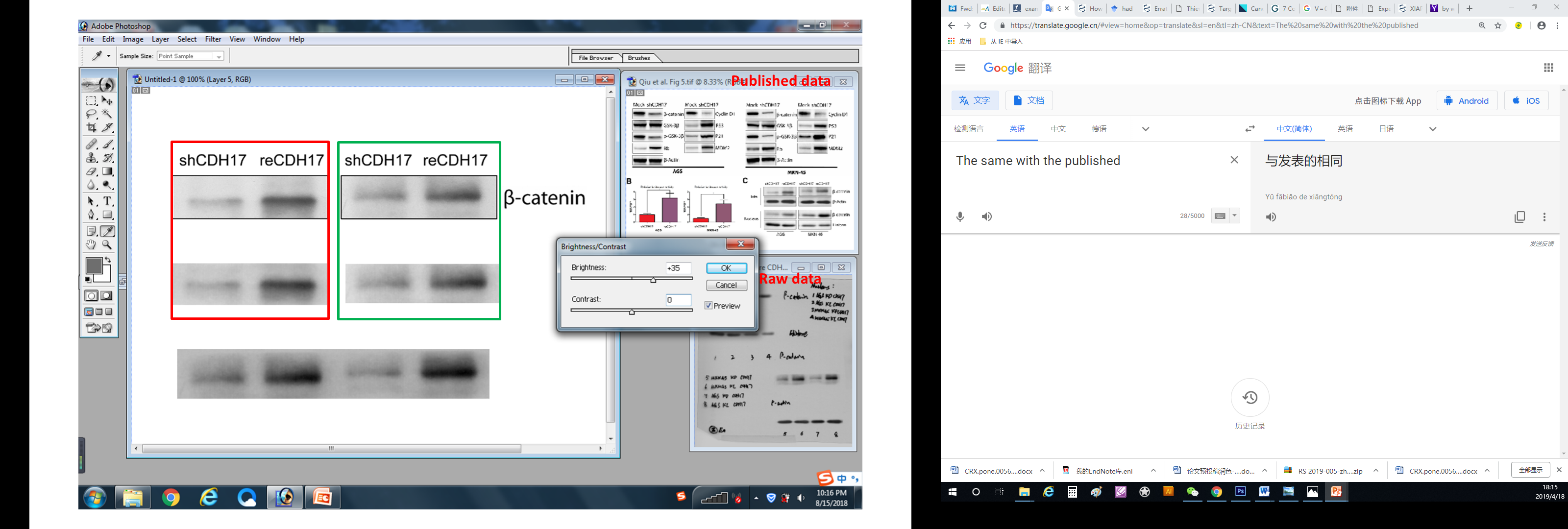

Supplement: S3 File — (DOCX) [file pone.0217124.s003.docx]
